# DietaryMineral Density and Clinical Outcomes in Adults with Long COVID: A Cross-Sectional Analysis of Global and Sex-Specific Associations in the BioICOPER Study

**DOI:** 10.3390/nu18203383

**Published:** 2026-10-15

**Authors:** Marta Gómez-Sánchez, David Rodríguez García, Elena Navarro Matías, Alicia Navarro-Cáceres, Silvia Arroyo-Romero, Nuria Suárez-Moreno, Andrea Domínguez-Martín, Manuel A. Gómez-Marcos, David Arjol, Leticia Gómez-Sánchez, BioICOPER Investigators Group

**Affiliations:** 1 Servicio de Hospitalización a Domicilio, Hospital Universitario Marqués de Valdecilla, 39008 Santander, Spain; martagmzsnchz@gmail.com; 2 Unidad de Investigación en Atención Primaria de Salamanca (APISAL), Gerencia de Atención Primaria de Salamanca, Gerencia Regional de Salud de Castilla y León (SACyL), Avenida de Portugal 83, 37005 Salamanca, Spain; drodriguezgarc@saludcastillayleon.es (D.R.G.); enavarro@saludcastillayleon.es (E.N.M.); alicia.nav@usal.es (A.N.-C.); 3 Instituto de Investigación Biomédica de Salamanca (IBSAL), Paseo de San Vicente, 58-182, 37007 Salamanca, Spain; silvia_ar@usal.es (S.A.-R.); nuria.suarez@usal.es (N.S.-M.); andreadm@usal.es (A.D.-M.); 4 Departamento de Medicina, Universidad de Salamanca, Calle Alfonso X el Sabio s/n, 37007 Salamanca, Spain; 5 Red de Investigación en Cronicidad, Atención Primaria y Promoción de la Salud (RICAPPS), Avenida de Portugal 83, 37005 Salamanca, Spain; 6 Servicio de Urgencias, Hospital Universitario Príncipe de Asturias Alcalá, Campus Exterior de la Universidad de Alcalá, Carretera de Meco, s/n, 28805 Alcalá de Henares, Spain; leticiagmzsnchz@gmail.com

**Keywords:** long COVID, post-COVID-19 condition, dietary minerals, micronutrients, mineral density, depression, nutritional epidemiology, sex differences

## Abstract

**Background/Objectives:** The role of routine dietary mineral intake in the clinical expression of long COVID is poorly studied. The association between the energy-adjusted dietary density of nine minerals and 16 clinical outcomes was analyzed, exploring differences according to sex. **Methods:** Cross-sectional study in 290 participants of the BioICOPER study (93 men and 197 women). Intake was estimated with a validated seven-day record; calcium, iron, iodine, magnesium, zinc, selenium, sodium, potassium and phosphorus were expressed per 1000 kcal. Spearman correlations were performed with Benjamini–Hochberg correction (FDR), multivariable linear regression, bootstrap with 5000 resamplings and analysis by sex. **Results:** In the overall cohort (*n* = 290), bivariate correlations between dietary mineral densities and clinical outcomes were weak (|r| < 0.15), and none of the 144 tested associations survived false discovery rate (FDR) adjustment (q > 0.05). In exploratory multivariable regressions, higher iron and zinc densities were nominally associated with lower depressive symptoms. Formal mineral × sex interaction tests were restricted to k = 2 pairs that met screening criteria (iron and zinc with depressive symptoms); both were nominally significant (*p* bootstrap = 0.041 and 0.015, respectively) without correction for multiplicity, indicating tentative, exploratory findings. **Conclusions:** No robust global associations were observed between dietary mineral density and clinical burden. The identified associations, especially the relationship between zinc and depressive symptoms in women, are exploratory and require prospective confirmation.

## 1. Introduction

Long COVID is defined as symptoms persisting for at least 2 months, typically starting within 3 months of SARS-CoV-2 infection, without alternative diagnosis [1]. This heterogeneous, multisystemic condition commonly presents with fatigue, dyspnea, cognitive and sleep disturbances, and functional limitations, alongside potential cardiovascular, autonomic, gastrointestinal, and psychological manifestations [2,3]. Its pathophysiology integrates overlapping mechanisms including viral persistence, immune dysfunction, sustained inflammation, endothelial and coagulation alterations, mitochondrial dysfunction, and microbiome shifts [3,4]. This broad clinical phenotype requires a multidimensional assessment capturing symptomatic burden alongside physical, cognitive, and emotional consequences [5,6]. Notably, female sex is consistently associated with higher long COVID risk [6], and previous BioICOPER cohort findings demonstrate a marked coexistence of physical, cognitive, and psychological manifestations [7]. These observations support both a comprehensive evaluation and explicit consideration of sex differences. Among modifiable factors, nutritional status and diet have gained attention. Appetite loss and taste/smell disturbances can impair dietary intake, while malnutrition risk is linked to higher symptom burden [8,9]. However, evidence regarding nutrition in long COVID remains fragmented, with heterogeneous study designs and a heavy focus on supplementation [10,11]. Recently, poorer diet quality was associated with greater symptom burden—particularly regarding fatigue, memory, and mental health—especially in women, though cross-sectional designs preclude establishing causality [12].

Minerals play essential roles in energy metabolism, neuromuscular excitability, neurotransmission, antioxidant defense, immune function, and erythropoiesis. However, long COVID evidence relies mainly on serum levels or interventions rather than usual dietary intake. Lower serum magnesium correlates with persistent symptoms [13] and higher symptom frequency when combined with vitamin D deficiency [14], while magnesium plus vitamin D supplementation reduced depressive symptoms in a small open-label trial [15]. Additionally, hypozincemia occurs in long COVID [16], and altered iron homeostasis and inflammatory erythropoiesis are linked to recovery and symptom burden [17,18]. Persistently altered metallomic profiles [19], increased fatigue from low selenium coexisting with SELENOP autoantibodies [20], and lower serum calcium have also been reported [21]. While providing biological plausibility, these findings do not prove that insufficient dietary intake causes symptoms or that supplementation yields clinical benefit. Consequently, information on the relationship between routine intake of a broad panel of minerals and a multidimensional set of clinical manifestations in adults with long COVID remains limited. The BioICOPER study was designed to characterize the vascular and biopsychological situation of this population in an integrated manner [22]. In this cohort, previous work assessed the dietary density of nine minerals and different indicators of vascular health, incorporating sex-specific analyses [23]. However, it remains unclear whether these dietary exposures are associated with global symptom burden, sleep, fatigue, frailty, cognition, mental health, pain, dyspnea, and quality of life. The choice of dietary mineral density (intake per 1000 kcal) over absolute intake was made because adjusting for energy intake controls confounding due to total food volume, which fluctuates significantly in Lon COVID due to appetite changes and anosmia.

We have condensed the general narrative on Long COVID clinical manifestations and mineral biology to ensure a sharper focus on our research scope. Furthermore, we have explicitly justified the choice of dietary mineral density (intake per 1000 kcal) over absolute intake: adjusting for energy intake controls for confounding due to total food volume (which fluctuates significantly in Long COVID due to appetite changes and anosmia) and reflects overall dietary nutritional quality independent of individual caloric requirements, enabling robust and unbiased comparisons across sexes.

Accordingly, the primary objective of this cross-sectional study was to investigate the global associations between habitual dietary density of nine key minerals (calcium, iron, iodine, magnesium, zinc, selenium, sodium, potassium, and phosphorus) and 16 multidimensional clinical outcomes in adults with Long COVID from the BioICOPER study. As a secondary exploratory objective, we evaluated whether these potential associations differed by sex through sex-stratified analyses and explicit interaction testing. We hypothesized that higher dietary mineral density would be associated with a lower clinical symptom burden, while recognizing the exploratory nature of these relationships given the clinical heterogeneity of the condition and the multiple exposure-outcome comparisons evaluated.

## 2. Materials and Methods

### 2.1. Study Design and Participants

An observational, cross-sectional, and analytical study was conducted from the BioICOPER cohort. The original protocol envisaged the inclusion of 300 participants [22] and the cohort ultimately consisted of 304 adults with Long COVID. For the present secondary analysis, an analytical sample of 290 participants (93 men and 197 women) was used with valid dietary information to calculate the density of the nine minerals studied and with the necessary data for the planned analyses. This sample was used both in the descriptive analyses and in the association analyses between dietary mineral density and clinical manifestations. The study was carried out at the Salamanca Primary Care Research Unit (APISAL). The BioICOPER project was registered in ClinicalTrials.gov on 18 April 2023 (NCT05819840), and its protocol has been previously published [22].

Patients from primary care and hospital settings were recruited through consecutive sampling during 2023. Adults who met the World Health Organization’s definition of long COVID were eligible: a history of probable or confirmed SARS-CoV-2 infection, presence of symptoms usually from 3 months after the onset of infection, persistence for at least 2 months, and absence of an alternative diagnosis to explain them [1]. All participants were required to provide written informed consent. Excluded were people with terminal illness, inability to go to the health center and complete the assessment, history of cardiovascular disease (including ischaemic heart disease or cerebrovascular disease), or an estimated glomerular filtration rate of less than 30 mL/min/1.73 m^2^.

**Figure 1:**
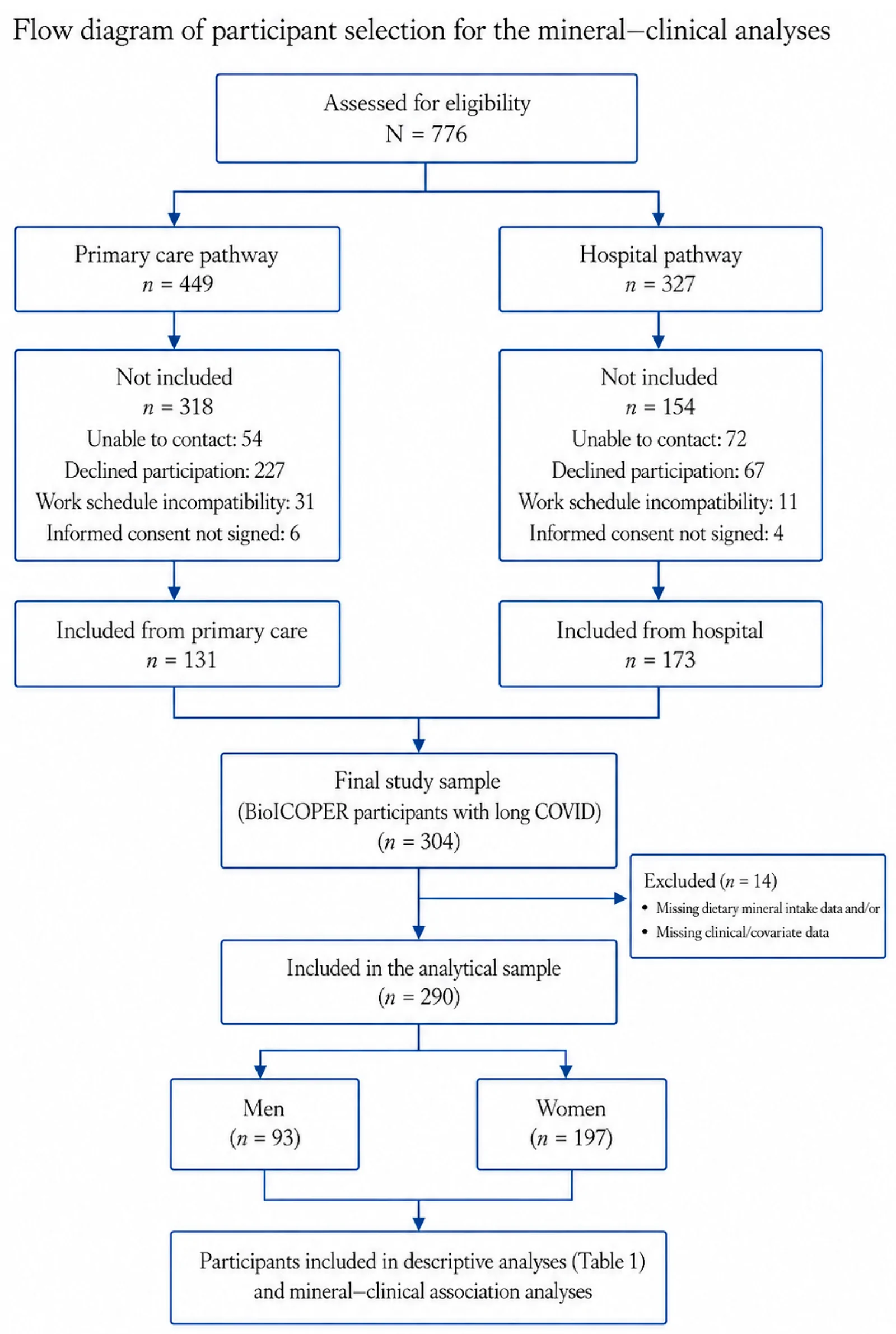
Flowchart of the selection of participants for mineral-clinical analyses. The diagram shows the recruitment process of the BioICOPER cohort, the exclusions during the selection and the collection of the final analytical sample used in the descriptive analyses and in the association analyses between dietary mineral density and clinical manifestations.

The recruitment process, exclusions, and obtaining the final analytical sample are summarized in Figure 1. The study was drafted following the recommendations of Strengthening the Reporting of Observational Studies in Epidemiology (STROBE) for observational studies [24]; the completed checklist is included as Supplementary Table S1.

### 2.2. Data Collection and Quality Control

The evaluations and collection of the variables were carried out by previously trained health personnel, following a standardized protocol. The information was recorded and managed using Research Electronic Data Capture (REDCap), hosted at the Instituto de Investigación Biomédica de Salamanca (IBSAL). An independent researcher carried out the quality control of the information collected [22].

### 2.3. Assessment of Dietary Intake

Dietary intake was assessed using a seven-day food and beverage registry carried out with the EVIDENT mobile application [25]. This tool was developed and validated by the Primary Care Research Group of Castilla y León, belonging to the Research Network on Preventive Activities and Health Promotion (REDIAPP), and is registered under intellectual property number 00/2014/2207. During the registration period, participants collected all food and beverages consumed and their corresponding portion sizes on a daily basis. The foods were assigned to the predefined groups of the application. Based on the composition data of Spanish foods incorporated in EVIDENT, the mean daily intake of iron (Fe), magnesium (Mg), selenium (Se), iodine (I), calcium (Ca), phosphorus (P), sodium (Na), potassium (K) and zinc (Zn) was calculated [25]. Discretionary salt added during cooking or at the table was not individually quantified; thus, sodium and iodine estimations reflect intrinsic food content, standard recipe formulations, and fortified foods selected by participants. Dietary records were evaluated for energy intake plausibility (daily energy intakes <800 kcal/day or >4000 kcal/day in men, and <500 kcal/day or >3500 kcal/day in women). Implausible energy records were excluded from the final analysis. Dietary mineral densities were calculated by dividing the absolute daily intake of each mineral by total energy intake (kcal/day) and multiplying by 1000, expressed as mg/1000 kcal or µg/1000 kcal. The use of the nutrient density method (expressed per 1000 kcal) is based on standard methodological recommendations in nutritional epidemiology. This nutrient density approach isolates overall diet quality from total food volume and mitigates confounding by individual energy requirements and illness-related changes in appetite, reducing the variability of caloric intake between people and ignoring differences due to body size, physical activity or loss of appetite associated with the disease. In multivariable regression models, total daily energy intake was also included as an additional covariate to control for potential residual energy confounding. Importantly, dietary assessments evaluated food-derived nutrient intakes exclusively; data on dietary supplement use and composition were not available and thus could not be integrated into total mineral exposure estimates.

Adequacy of intake was assessed with the absolute daily average intake, and not with energy density variables. The cut-off points were established based on the dietary reference values of the European Food Safety Authority (EFSA) [26] and Spanish Federation of Nutrition, Food and Dietetic Societies (FESNAD) [27]. When differentiated values existed, sex-specific cut-off points were applied. The actual menopausal status of women was not available in our database. We considered women who were >50 years of age as menopausal and premenopausal women who were ≤50 years of age. As cut-off points for iron, we used 18 mg/day for women who were ≤50 years and 10 mg/day for those who were >50 years old and men. Values below these reference thresholds were interpreted as intakes below recommended levels or risk of inadequate intake, rather than clinical or biochemical deficiency. For all minerals except sodium, an average intake equal to or greater than the reference value was classified as adequate compliance; lower intakes were considered inadequate or at risk of insufficiency. In the case of sodium, compliance corresponded to maintaining intake below 2000 mg/day. The specific cut-off points for each mineral are detailed in Supplementary Table S2.

The dietary record did not quantify the nutrients provided by supplements. Therefore, both mineral densities and adequacy classification represent only the estimated intake from the recorded foods and beverages and not the total exposure from foods plus supplements.

### 2.4. Clinical Variables and Comprehensive Assessment of Symptoms

Clinical variables were obtained through review of the clinical history, structured history, and validated questionnaires, following the BioICOPER study protocol [22]. History of SARS-CoV-2 infection, number and date of infections, diagnostic method, hospitalization during the acute phase, and time from infection to evaluation were recorded. Symptomatic burden was assessed by recording persistent manifestations grouped into six domains: (1) systemic, which included fatigue, lack of energy, and fever; (2) neurocognitive, which included memory loss, difficulty concentrating, and brain fog or confusion; (3) respiratory or cardiovascular, which included dyspnea, cough, sore throat, and weight loss; (4) musculoskeletal, which included muscle pain, joint pain, and reduced mobility; (5) neurological or neuromuscular, which included headache, taste or smell disturbances, and decreased reflexes; and (6) psychosocial or psychiatric, which included depression, anxiety, and sleep disturbances [22]. For the present analysis, the global symptom burden was expressed as the number of domains in which the participant had at least one persistent manifestation, with a possible score of 0–6. The impact of fatigue during the previous four weeks was assessed using the Modified Fatigue Impact Scale (MFIS) [28]. This scale contains 21 items scored from 0–4 and provides scores in three dimensions: physical, consisting of 9 items and with a range of 0–36 points; cognitive, consisting of 10 items and with a range of 0–40 points; and psychosocial, consisting of 2 items and with a range of 0–8 points. The total score ranges from 0–84 points; higher scores indicate a greater impact of fatigue. Sleep quality during the previous month was assessed using the Spanish version of the Pittsburgh Sleep Quality Index (PSQI) [29,30]. Its items generate seven components: subjective sleep quality, latency, duration, habitual efficiency, sleep disturbances, use of hypnotic medication, and daytime dysfunction. The sum of these components provides an overall score of 0 to 21 points, with higher scores indicating poorer sleep quality. Perceived cognitive failures in daily activities were assessed using the 30-item Memory Failures of Everyday questionnaire (MFE-30), validated in the Spanish population [31]. Each item captures the frequency of a memory failure through five response categories, from “never or almost never” to “always or almost always”. The total score of the scale was used, so that higher values represent a higher frequency of subjective memory failures. Dyspnea was assessed using the modified Medical Research Council (mMRC) scale [32]. This scale classifies dyspnea-related functional limitation between 0 and 4 points, ranging from the appearance of dyspnea only during intense exercise to the inability to leave the home or the presence of dyspnea during basic activities such as dressing. Higher scores indicate greater dyspnea limitation. Pain was assessed using the Spanish version of the Graded Chronic Pain Scale or Chronic Pain Grade Questionnaire [33]. The instrument consists of eight items that assess the presence and intensity of pain and its interference with daily, work, and leisure activities during the previous three months. In this study, the continuous pain score was used, so that higher values indicate greater intensity or functional impact. Health-related quality of life was assessed using the Spanish version of the Short Form-36 Health Survey (SF-36) [34]. The questionnaire contains 36 items distributed in eight dimensions: physical function, physical role, bodily pain, general health, vitality, social function, emotional role, and mental health. For this analysis, the Physical Component Summary (PCS), the Mental Component Summary (MCS), and the Mental Health (MH) dimension score were used. In these variables, a higher score represents better health status. Frailty was assessed using the FRAIL scale [35], composed of five dichotomous items that assess fatigue, endurance, aerobic capacity, comorbidity, and weight loss. The total score ranges from 0 to 5 points and allows participants to be classified as robust (0 points), prefragile (1–2 points), or fragile (3–5 points). The total score of the scale was used in the analyses. Symptoms of anxiety and depression were assessed using the Spanish version of the Goldberg Anxiety and Depression Scale (GADS) [36,37]. The instrument includes two subscales of nine items: anxiety and depression. Each affirmative answer adds one point. Scores ≥ 4 on the anxiety subscale and ≥2 on the depression subscale are considered indicative of possible clinical alteration. However, the original scores of both subscales were used in the association analyses, without dichotomizing them. Objective cognitive function was assessed using the Montreal Cognitive Assessment (MoCA), using its validated version in Spanish [38,39]. The MoCA examines attention and concentration, executive functions, memory, language, visuoconstructive skills, conceptual reasoning, calculation, and orientation. The maximum score is 30 points and a score ≥26 is considered indicative of normal cognitive performance. The continuous total score was used in the analyses, where higher values represent better cognitive performance. All scales were analysed using their original scores. Ordinal variables or variables with non-normal distribution were described using median and interquartile range. In interpretive terms, higher scores in PSQI, MFIS, MFE-30, FRAIL, GADS, pain, and mMRC indicate a more unfavorable clinical situation, while higher scores in SF-36 and MoCA indicate better quality of life and cognitive performance, respectively.

### 2.5. Covariates and Other Study Variables

Age, sex, body mass index, hypertension, diabetes mellitus, dyslipidemia, current smoking, alcohol consumption, daily steps, physical activity expressed in MET-min/week, sedentary time and adherence to the Mediterranean diet were recorded using the MEDAS score, in addition to hospitalization during the acute phase of COVID-19 and the time elapsed since infection. These variables were obtained following the standardized protocol of the BioICOPER study and were used to characterize the sample. For the multivariate models, age, sex, time since infection, body mass index and hospitalization during the acute phase were selected a priori for their clinical relevance and their possible relationship with dietary exposure and the results were analyzed.

### 2.6. Statistical Analysis

Analyses were performed on the analytical sample of 290 participants with long COVID who had valid mineral intake information and the clinical and covariate data required for each analysis. All tests were bilateral. A *p* value of <0.05 was considered statistically significant in the analyses not subject to multiplicity correction and a q-value < 0.05 in the Benjamini–Hochberg corrected correlation families. *p* values were presented to three decimal places and as *p* < 0.001 when they were below that threshold. Statistical analyses were performed using IBM SPSS Statistics 32 (IBM Corp., Armonk, NY, USA). The main exposures were dietary intakes of calcium, iron, iodine, magnesium, zinc, selenium, sodium, potassium and phosphorus, expressed as energy density per 1000 kcal. In addition, compliance with intake recommendations was classified using previously defined cut-off points, specific by sex when appropriate in the dietary assessment section.

The distribution of continuous variables was assessed using the asymmetry and kurtosis coefficients and the Kolmogorov–Smirnov tests with Lilliefors correction and Shapiro–Wilk correction. Age and potassium density showed an approximately normal distribution and were summarized by mean and standard deviation. The rest of the continuous variables or bounded scores were described by median and interquartile range, while categorical variables were expressed as absolute frequency and percentage. The modified Medical Research Council (mMRC) dyspnea score, due to its ordinal nature, was described by median and interquartile range, and was not included in the normality tests, as detailed in Supplementary Table S3. The characteristics of men and women were compared using the Student’s *t*-test for independent samples when the variable had an approximately normal distribution and using the Mann–Whitney U test for non-normal or ordinal variables. Categorical variables were compared using Pearson’s chi-square test; Fisher’s exact test was used when some expected frequency was less than 5. The same chi-square procedure was used to compare between sexes the percentages of participants who met the intake recommendations for each mineral.

Baseline associations between dietary mineral density and clinical features were examined using Spearman’s rank correlation coefficient (ρ), due to the predominantly non-normal distribution of variables. Nine minerals were analyzed against 16 clinical outcomes: number of persistent symptom domains; Pittsburgh Sleep Quality Index (PSQI) total score; physical, cognitive, and psychosocial subscales and Modified Fatigue Impact Scale (MFIS) total score; FRAIL score; 30-item Memory Failures of Everyday Questionnaire (MFE-30) total score; Montreal Cognitive Assessment (MoCA); physical and mental health components and mental health domain of the 36-Item Short Form Health Survey (SF-36); anxiety and depression subscales of the Goldberg Anxiety and Depression Scale (GADS); pain score of the Chronic Pain Grade Questionnaire (CPGQ); and mMRC dyspnea score. In the global sample, 144 correlations were obtained (9 minerals × 16 results). To control for multiplicity, the *p*-values were adjusted using the Benjamini–Hochberg procedure, considering the 144 correlations as a single test family. Subsequently, the correlations were repeated separately in males and females; in this stratified analysis, the Benjamini–Hochberg correction was applied jointly to the 288 resulting correlations (9 minerals × 16 outcomes × 2 sexes). A value of q < 0.05 was considered statistically significant. Associations with *p* < 0.05 but q ≥ 0.05 were described exclusively as nominal.

Mineral-outcome pairs that showed a nominal association in the global screening were subsequently studied using exploratory multiple linear regression models. Each mineral-outcome combination was analyzed in an independent model, introducing mineral density as a continuous variable in its original units (mg/1000 kcal or μg/1000 kcal, depending on the mineral). Model 1 was adjusted for age and sex. Model 2 aggregated time since SARS-CoV-2 infection, body mass index, and hospitalization during the acute phase of COVID-19. These covariates were selected for their clinical relevance and for their potential relationship with dietary exposure and clinical outcomes. Non-standardized regression coefficients (B), their 95% confidence intervals, and *p*-values were presented. Each model was estimated using complete case analyses for the outcome and included covariates. The stability of the selected Model 2 associations was assessed using non-parametric bootstrap with 5000 replacement resamplings. Bootstrap bias, standard error, and bias-corrected and accelerated (BCa) 95% confidence intervals were calculated. An association was considered to be supported in the sensitivity analysis when the BCa interval did not include the null value. Since the models were pre-screened based on nominal significance and no overall correlation exceeded the false-discovery rate correction, these analyses were interpreted as exploratory. It is important to emphasize that bootstrapping evaluates internal parameter stability and sampling variability under non-normality within the specified models; it does not adjust for the multiplicity of the initial screening step (144 correlation tests) nor does it correct for post-selection inference bias. Therefore, bootstrap intervals should be interpreted strictly as measures of internal model stability rather than confirmatory validation of true population-level associations.

In addition to the stratified correlations, the modification of the effect by sex was formally evaluated by incorporating an interaction term between mineral density and sex into the fully adjusted models. To facilitate interpretation and reduce the collinearity associated with the product term, the mineral densities used in the interaction models were transformed into z-scores, with mean 0 and standard deviation 1. Sex was coded using males as a reference category; therefore, the coefficient of the mineral term × sex represented the difference between the estimated slope in females and the estimated slope in males for each one-standard-deviation increment in mineral density. For each interaction, the specific adjusted slopes of males and females, with their 95% confidence intervals, were estimated, and the non-interaction model was compared with the model that included the product term. The additional contribution of the interaction was quantified by the change in the coefficient of determination (ΔR^2^) and its test of change. Collinearity was examined using the variance inflation factor (VIF). Likewise, the interaction coefficients were bootstrapped with 5000 resamplings and calculation of 95% BCa intervals. The interaction *p*-values were interpreted as nominal, since adjusted q-values were not available for the complete family of planned exploratory interactions. Consequently, even if the BCa interval excluded the null value, the modification by sex was considered an exploratory and not confirmatory finding. Formal testing of mineral × sex interaction terms was restricted exclusively to the k = 2 mineral–outcome pairs (iron–depression and zinc–depression) that met the pre-specified criteria in the initial screening. No other mineral–outcome combination fulfilled the selection threshold for multivariable interaction modeling. Because interaction *p*-values were not adjusted for multiplicity, they were interpreted as strictly nominal and exploratory.

To evaluate model assumptions and ensure that results were not driven by influential observations or the bounded, discrete nature of the GADS depression score (0–9), Cook’s distance [with a cut-off threshold of 1.0] was calculated, and sensitivity analyses were performed using ordinal logistic regression (PLUM) for the fully adjusted models.

To assess whether the associations of iron and zinc with depressive symptoms in women were mutually independent, a secondary analysis restricted to the 197 women in the sample was performed. First, the correlation between iron and zinc densities was quantified using Spearman. Subsequently, both densities, standardized as z-scores, were simultaneously entered into a linear model adjusted for age, time since SARS-CoV-2 infection, body mass index, and hospitalization during the acute phase of COVID-19. The collinearity between the two minerals was assessed using VIF. The additional variance explained by the joint incorporation of iron and zinc was estimated using ΔR^2^ with respect to the model containing only the covariates. Finally, the coefficients of both minerals were evaluated by bootstrap with 5000 resamplings and 95% BCa intervals. To address potential residual confounding by overall diet quality, total dietary volume, lifestyle, and concomitant therapy, an expanded multivariable sensitivity model (Model 3) was fitted. This model additionally adjusted Model 2 covariates for Mediterranean diet adherence (MEDAS score), total energy intake (kcal/day), alcohol consumption (g/week), and baseline use of antidepressant medication.

### 2.7. Ethical Considerations

The study was approved on 27 June 2022 by the Ethics Committee for Research with Medicines of the Salamanca Health Area (CEIm; Ref. PI 2022 06 1048). It was carried out in accordance with the ethical principles of the Declaration of Helsinki in its current version. The confidentiality of the participants was guaranteed in accordance with Organic Law 3/2018 and Regulation (EU) 2016/679 on data protection. All participants gave written informed consent prior to their inclusion, after receiving detailed information about the study procedures.

### 2.8. Use of Artificial Intelligence Tools

During the preparation of the manuscript, ChatGPT 5.6 (OpenAI) was used: https://chatgpt.com to support linguistic review, editorial coherence, and figure layout from author-generated results. Generative artificial intelligence was not used to generate data, perform statistical analysis, create original scientific images, or modify the results of the study. The authors compared the AI-assisted figures and text with the original statistical data and results, reviewed and approved all modifications, and took full responsibility for the final content of the manuscript.

## 3. Results

### 3.1. Study Participants

The analytical sample included 290 participants with long COVID, 93 men (32.1%) and 197 women (67.9%). Assessment of distribution showed that most clinical, lifestyle, and dietary variables deviated from normal, while age and potassium density had an approximately normal distribution. Consequently, approximately normal variables were summarized by mean ± standard deviation and the rest of the continuous variables or scores were bounded by median [interquartile range]. This pattern also supported the use of nonparametric methods for the initial screening of mineral-clinical associations (Supplementary Table S3).

As shown in Table 1, men were older and had a higher body mass index than women. Hospitalization during the acute phase of COVID-19, hypertension, diabetes mellitus, and dyslipidemia were also more common in men, and alcohol consumption was higher. In contrast, no significant differences were observed between sexes in the time since SARS-CoV-2 infection, current smoking, daily steps, physical activity, sedentary time, adherence to the Mediterranean diet, or total energy intake. Dietary mineral densities were generally comparable between sexes. (Table 1). The proportions of participants who met the recommended intake criteria for each mineral, in the overall sample and by sex, are shown in Supplementary Figure S1.

We observed no differences between the 290 included subjects and the 14 non-included subjects, except in age (Supplementary Table S4).

### 3.2. Clinical Characteristics According to Sex

Clinical features are summarized in Table 2. Women had a higher overall burden of persistent symptoms, poorer sleep quality, and a greater impact of fatigue on physical, cognitive, psychosocial, and total MFIS scores. They also showed higher FRAIL scores, more subjective memory lapses, higher anxiety and depression scores, and increased pain. Accordingly, men had higher scores on the physical and mental summary components and the mental health domain of the SF-36, indicative of better perceived health status. Dyspnea also differed by sex, with greater limitation in women. In contrast, the objective cognitive performance assessed by the Montreal Cognitive Assessment was comparable between men and women (*p* = 0.116). Together, these results show a higher subjective, functional, and psychoaffective clinical burden in women with long COVID (Table 2).

### 3.3. Global Associations Between Dietary Mineral Density and Clinical Outcomes

**Figure 2:**
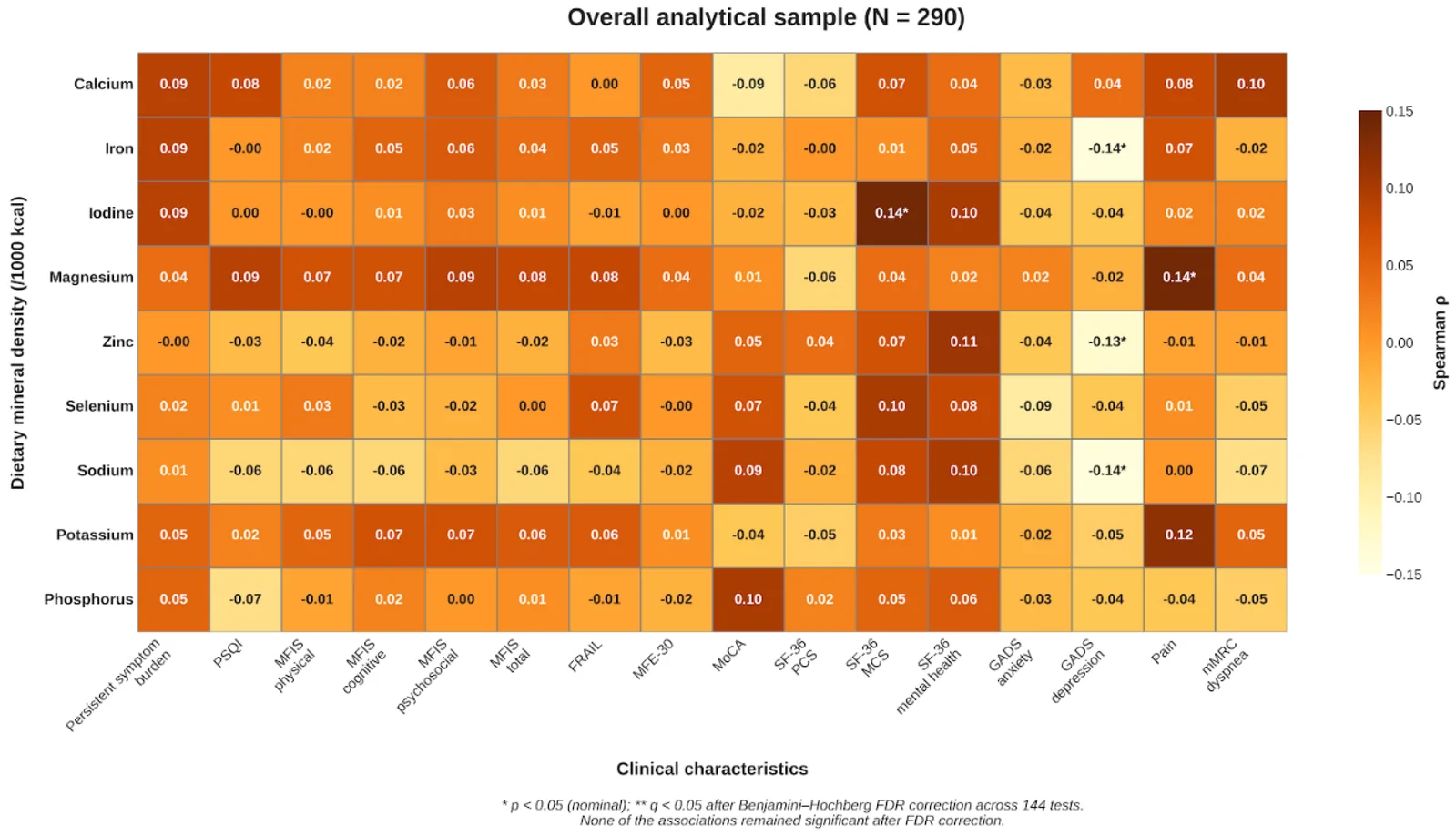
Spearman correlations between dietary mineral density and clinical outcomes in participants with long COVID. The heat map shows the correlation coefficients by Spearman ranges (ρ) between dietary mineral density, expressed per 1000 kcal, and clinical, cognitive, functional, psychological, and health-related quality of life measures in the global analytical sample. Positive values indicate direct associations and negative values indicate inverse associations. An asterisk (*) indicates nominal significance with *p* < 0.05 and two asterisks (**) indicate significance after the Benjamini–Hochberg correction for the 144 correlations. No association remained significant after FDR correction. Sample size varied slightly between clinical outcomes due to missing data.

In the primary global analysis encompassing the entire cohort (*n* = 290), bivariate correlations between dietary mineral densities and clinical outcomes were generally weak (|*r*| < 0.15). Following Benjamini–Hochberg false discovery rate (FDR) adjustment across all 144 tested pairs, no global association survived correction for multiple comparisons (*q* > 0.05 for all). Prior to FDR adjustment, five nominally significant associations were identified: higher iron, zinc, and sodium densities were associated with lower GADS depression scores; higher iodine density was associated with a higher mental component score of the SF-36; and higher magnesium density was associated with a higher pain score. (Figure 2; Supplementary Table S5).

**Figure 3:**
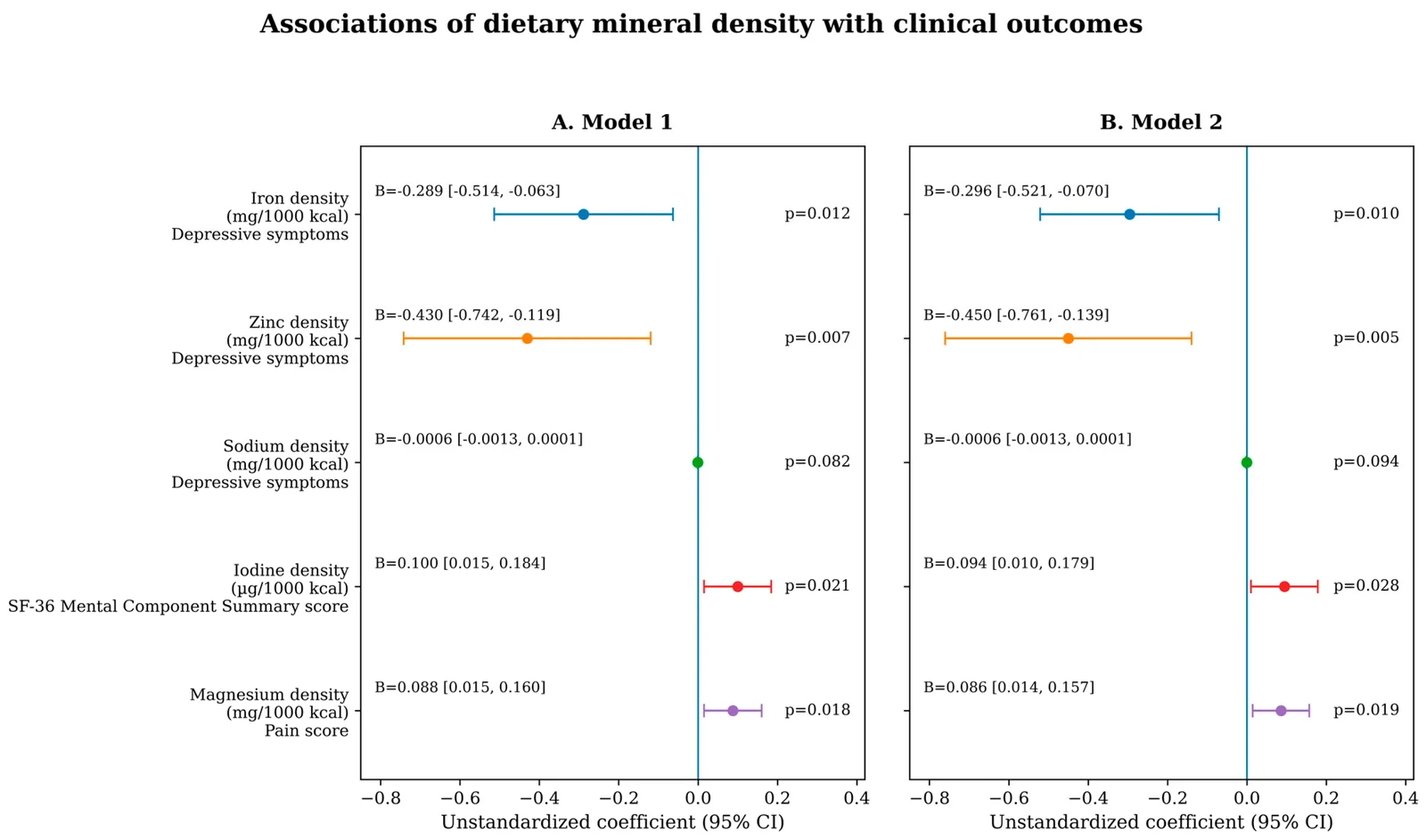
Associations between dietary mineral density and clinical outcomes in adjusted linear regression models. Non-standardized regression coefficients (**B**) and their 95% confidence intervals are shown for the five mineral-clinical pairs selected after nominal screening. Model 1 was adjusted for age and sex; Model 2 added time since SARS-CoV-2 infection, body mass index, and hospitalization during the acute phase. Associations with iron, zinc, iodine, and magnesium maintained a similar direction and magnitude after additional adjustment, while the confidence interval of the sodium-depression association included the null value. These analyses are exploratory because no overall correlation exceeded FDR correction.

The five mineral-clinical pairs with nominal significance were subsequently examined using exploratory adjusted linear regression models (Figure 3). Model 1 was adjusted for age and sex, and Model 2 added time since SARS-CoV-2 infection, body mass index, and hospitalization during the acute phase. The magnitude and direction of the estimates changed little after further adjustment. Higher iron and zinc densities were associated with lower depressive symptom scores in both models. Higher iodine density was associated with a higher mental component score of the SF-36 and higher magnesium density with a higher pain score. The inverse association between sodium density and depressive symptoms did not reach statistical significance in either model (Figure 3). The results were not altered by additional adjustment for total energy intake (Figure S3).

### 3.4. Sex-Specific Mineral-Clinical Associations

Sex-stratified correlation analyses showed a different pattern (Supplementary Figure S2). Few nominal associations were observed in men, and none remained significant after FDR correction. A higher number of nominal associations were identified in women, especially for psychological and quality of life outcomes. After applying the Benjamini–Hochberg correction together to the 288 stratified tests, only two associations remained statistically significant, both in women: iron density with depressive symptoms (ρ = −0.259; *p* < 0.001; q = 0.041) and zinc density with depressive symptoms (ρ = −0.256; *p* < 0.001; q = 0.041).

Formal interaction analyses were conducted for the k = 2 mineral–outcome pairs that reached nominal significance in the initial screening (Table 3). Interaction terms (mineral z-score × sex) were nominally significant for iron (B = −0.634, *p* bootstrap = 0.041; ΔR^2^ = 0.013) and zinc (B = −0.836, *p* bootstrap = 0.015; ΔR^2^ = 0.019) in relation to GADS depression scores. No other mineral–outcome pairs were tested for interaction, as none met the preliminary screening criteria. Given that this family of interaction tests was not corrected for multiple comparisons, these results should be interpreted as nominal, tentative, and hypothesis-generating.

**Figure 4:**
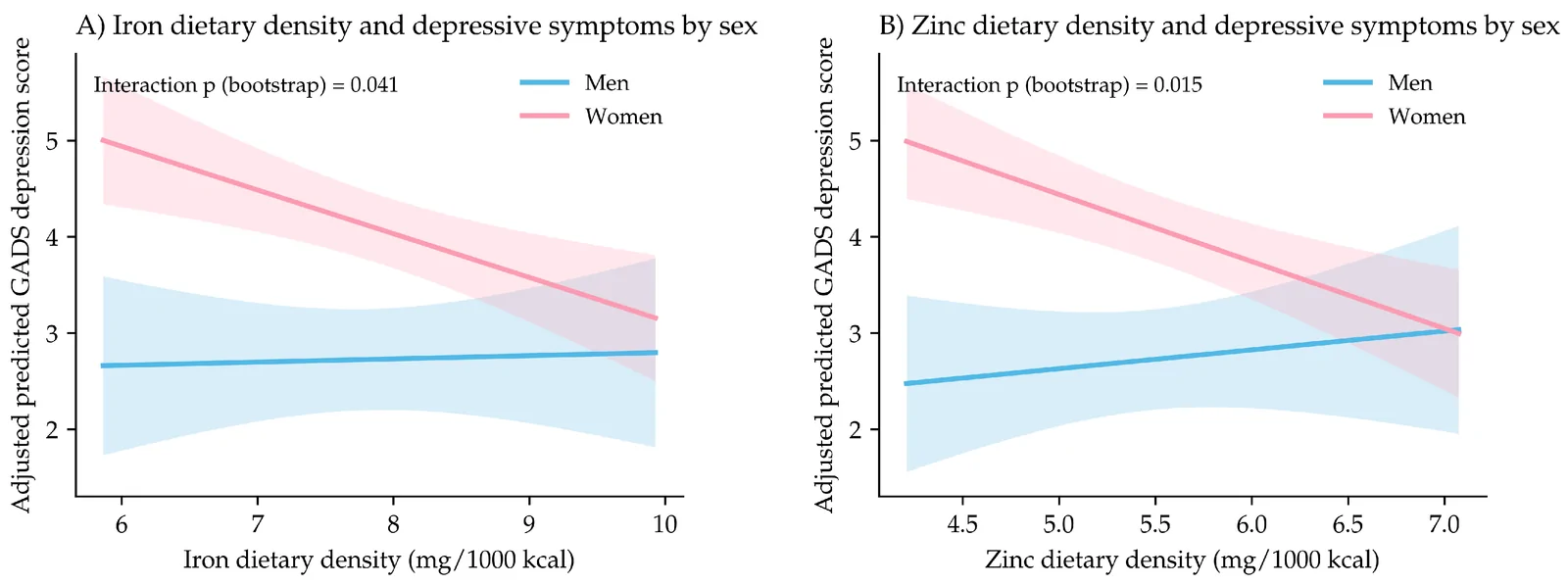
Adjusted associations of dietary iron and zinc densities with depressive symptoms by sex. Predicted GADS depression scores and their 95% confidence intervals are shown along the P5–P95 range of dietary density of (**A**) iron and (**B**) zinc in men and women. Models were adjusted for age, time since SARS-CoV-2 infection, body mass index, and hospitalization during the acute phase. Shaded areas represent 95% confidence intervals. Inverse associations were observed in females, and no significant associations were detected in males. Slope differences were nominal *p* bootstrap = 0.041 for iron and nominal *p* bootstrap = 0.015 for zinc; these interaction tests were not corrected for multiplicity.

Sex-specific adjusted associations are illustrated in Figure 4 using predicted GADS depression scores across the P5–P95 percentile range of dietary iron and zinc densities. In women, predicted depression scores decreased significantly with higher mineral density—associations that remained statistically significant even after rigorous correction for multiple testing across all stratified analyses. In contrast, adjusted slopes among men remained flat near zero. While formal multiplicative interaction analyses (mineral × sex) supported this differential pattern, these interaction results lacked adjustment for multiplicity and should therefore be interpreted strictly as exploratory and hypothesis-generating.

To evaluate whether the inverse associations of dietary iron and zinc densities with depressive symptoms in women persisted after accounting for their co-ingestion, both minerals were simultaneously included in a fully adjusted model. Predictors were standardized (expressed per 1 SD increment) to enable direct comparison of effect sizes. Iron and zinc densities showed a moderate correlation (Spearman’s *ρ* = 0.650; *p* < 0.001), but collinearity remained acceptable (VIF = 1.91 for both exposures). Upon mutual adjustment, zinc retained a statistically significant inverse association with depressive symptom scores (*B* = −0.498 per 1 SD increase; 95% CI: −0.968 to −0.028; *β* = −0.199; *p* = 0.038), whereas the association for iron was substantially attenuated and non-significant (*B* = −0.219 per 1 SD increase; 95% CI: −0.689 to 0.252; *β* = −0.087; *p* = 0.360). Joint incorporation of both micronutrients yielded a significant gain in explained variance (Δ*R*^2^ = 0.070; *p* = 0.001). Internal validation via 5000 bootstrap resamples confirmed the robustness of the zinc association (95% BCa CI: −0.938 to −0.039; *p* = 0.027), while the bootstrap interval for iron crossed the null value (95% BCa CI: −0.706 to 0.243; *p* = 0.367). These findings indicate substantial shared variation between both dietary exposures, demonstrating that the inverse association of zinc is independent of iron, whereas the initial iron association was largely confounded by zinc co-intake.

### 3.5. Sensitivity and Robustness Analysis

Internal stability was evaluated using bootstrap resampling with 5000 resamplings for the associations selected from the fully adjusted model (Supplementary Table S6). For iron density and depressive symptoms, the bootstrap estimate was B = −0.296 (95% BCa CI −0.506 to −0.078; *p* = 0.005), and for zinc, B = −0.450 (95% BCa CI −0.716 to −0.157; *p* = 0.001). For iodine density and the mental component of the SF-36, the bootstrap 95% BCa CI was 0.010 to 0.176 (B = 0.094; *p* = 0.025), and for magnesium density and pain score, 0.020 to 0.155 (B = 0.086; *p* = 0.010). In contrast, for the sodium–depression association, the bootstrap 95% BCa CI spanned zero (B = −0.00060; 95% BCa CI −0.00130 to 0.00008; *p* = 0.103) (Supplementary Table S5). The bootstrap 95% BCa CIs for the iron–zinc interaction terms also excluded zero (Table 3). However, since the interaction tests were not corrected for multiplicity, this result should be considered exploratory. Taken together, these internal stability metrics show bootstrap 95% BCa CIs excluding zero for the inverse associations of iron and zinc densities with depressive symptoms in women, whereas the remaining global associations require special caution because initial screening did not exceed FDR correction.

Furthermore, to account for the bounded and discrete distribution of the GADS depression score (0–9), multivariable ordinal logistic regression (PLUM) analyses were conducted across both Model 2 (standard adjustment) and Model 3 (expanded sensitivity adjustment including Mediterranean diet adherence, total energy intake, alcohol consumption, and baseline antidepressant medication use). Across both model tiers, higher dietary densities of zinc and iron were consistently and inversely associated with depression severity in the overall sample (*n* = 290; all *p* ≤ 0.004) and particularly in women (*n* = 197; all *p* ≤ 0.001), with goodness-of-fit tests confirming a statistically significant overall model fit (*χ*^2^ test *p* < 0.001). Conversely, no significant associations were observed in men across any of the adjusted models (*n* = 93; all *p* ≥ 0.543). Full unstandardized B coefficients, 95% confidence intervals, and *p*-values for both Model 2 and Model 3 are detailed in Supplementary Table S7.

## 4. Discussion

The present study examined the relationship between the energy-adjusted dietary density of nine minerals and a broad set of clinical, functional, cognitive, psychological, and quality-of-life outcomes in adults with long COVID. The main findings were threefold. First, the adequacy of intake was heterogeneous: it was more common for selenium, zinc, and iron and less common for iodine, calcium, phosphorus, potassium, and magnesium. For sodium, the cut-off point (<2000 mg/day) represents a maximum recommended intake; therefore, its low adequacy indicates that a significant proportion exceeded that limit, rather than having an insufficient intake. Iron adequacy was more common in men, while that of magnesium and zinc was higher in women. Second, the overall pattern of associations between minerals and clinical manifestations was weak. All Spearman coefficients had absolute values below 0.15 and none of the 144 correlations remained statistically significant after controlling for the false discovery rate (FDR). Consequently, the data do not provide robust evidence of an overall relationship between individual mineral density and the multidimensional clinical burden of long COVID, although they do not allow excluding small or specific associations in certain subgroups. Third, within this predominantly null pattern, exploratory signals appeared that maintained an association-compatible estimate after multivariable adjustment and in bootstrap analyses.

### 4.1. Primary Global Finding—Absence of Broad Associations Between Dietary Mineral Density and Clinical Burden

An important primary result of the present study is the predominance of null or very small magnitude associations. None of the overall mineral–clinical correlations remained significant after FDR correction, and no consistent patterns were observed for calcium, selenium, potassium, or phosphorus across the extensive battery of clinical outcomes evaluated. These negative results are highly informative: the biological plausibility derived from studies on circulating mineral concentrations does not necessarily imply that differences in usual dietary intake are directly related to symptom severity. To properly contextualize these findings, it is essential to distinguish between habitual dietary intake and systemic mineral status. Dietary intake represents nutritional exposure, while circulating or tissue concentrations also depend on intestinal absorption, inflammation, metabolic redistribution, renal function, hormonal regulation, and other disease-related processes. This distinction is especially critical in long COVID, as much of the available mineral literature is based on serum biomarkers rather than usual dietary intake [13,14,15,16,17,18,19,20,21]. For example, persistent fatigue after SARS-CoV-2 infection has been associated with the combination of low selenium or SELENOP concentrations and the presence of autoantibodies against SELENOP, rather than with a single isolated determinant of selenium [20]. Likewise, lower serum calcium concentrations have been described in people who maintained post-COVID symptoms [21]. These studies describe alterations in systemic mineral homeostasis and disease biology, but do not assess routine dietary exposure.

Furthermore, the heterogeneous pattern of mineral adequacy observed in BioICOPER does not appear to be unique to long COVID. In the ANIBES population-based study, a considerable proportion of the Spanish population had lower than recommended calcium and magnesium intakes, and insufficient zinc intake was also frequent [40,41]. Likewise, iron intakes were lower in women than in men, consistent with their higher requirements during certain life stages and their increased risk of inadequate intake [42]. Regarding iodine, a systematic review of European dietary surveys showed below-recommended intakes in a significant proportion of national studies, especially among adult women, highlighting the limited number of concentrated food sources [43]. Recent global estimates also place iodine, calcium, and iron among the micronutrients with the highest prevalence of inadequate intake, with relevant sex differences [44]. Therefore, the dietary pattern found in BioICOPER seems to reflect, at least partially, suboptimal intake patterns observed in the general population rather than specific characteristics of long COVID.

More generally, recent literature has begun to shift interest away from isolated nutrients toward global dietary patterns. Poorer overall diet quality has been linked to higher symptom burden in women with long COVID [12]. Likewise, a prospective study with 69,070 participants from the UK Biobank found that a higher empirical inflammatory dietary pattern score was associated with an increased risk of long COVID, while the dietary inflammatory index did not show an equivalent association [45]. Although the magnitude of the effect was modest, taken together, these observations suggest that the complete dietary matrix and nutrient interactions might better reflect the potential nutritional contribution to symptom persistence than studying individual minerals in isolation.

### 4.2. Comparatively Stronger Exploratory Signal—Dietary Zinc, Iron, and Depressive Symptoms in Women

The clinical finding with the greatest internal consistency was the inverse relationship between dietary densities of iron and zinc and the burden of depressive symptoms. Although these associations came from a global exploratory screening whose associations did not survive overall FDR correction, several elements provide consistency to the signal. The magnitude of the effects was maintained after adjusting for age, sex, time since SARS-CoV-2 infection, body mass index, and hospitalization during the acute phase, and BCa intervals obtained by bootstrap excluded the null value. Most importantly, in sex-stratified analyses, iron and zinc were the only mineral-clinical associations that remained statistically significant after FDR correction in women. However, our interaction results warrant a particularly cautious interpretation. Formal tests of mineral × sex interaction were performed for only k = 2 selected pairs and were not corrected for multiplicity. Furthermore, the smaller male sample size (*n* = 93 vs. *n* = 197 in women) limits statistical power to detect subtle interaction effects or male-specific associations, increasing the uncertainty of point estimates in men. Consequently, these sex differences should not be viewed as established sex-specific biological mechanisms, but rather as exploratory signals that require prospective confirmation in larger, sex-balanced cohorts.

This pattern possesses biological plausibility. Iron is involved in oxygen transport, mitochondrial metabolism, and enzymatic processes related to neurotransmitter synthesis; zinc is involved in synaptic signaling, neuroplasticity, antioxidant defense, and immune regulation. A meta-analysis of observational studies found that higher dietary intake of zinc and iron was associated with a lower risk of depression, with more consistent evidence for zinc [46]. A systematic review of interventions in premenopausal women described possible improvements in some mood and cognition outcomes, though trials were heterogeneous and small [47]. A recent review on trace elements in major depressive disorder also noted alterations in circulating zinc and iron [48]. In long COVID literature, hypozincemia has been described in approximately one-fifth of patients evaluated in a long COVID clinic, with fatigue being common among those with low zinc concentrations [16]. Alterations in iron homeostasis constitute an even more developed mechanistic line: persistent iron dyshomeostasis and anemia have been linked to poorer recovery after infection [17], while a longitudinal study identified a signature characterized by unresolved inflammation, anemia, reduced circulating iron, alteration of iron homeostasis pathways, and stress erythropoiesis among people who subsequently developed post-COVID condition [18].

The sex-specific nature of this signal constitutes another relevant aspect. Women in this cohort had greater sleep impairment, physical and cognitive fatigue, subjective memory failures, anxiety, depression, pain, and dyspnea, as well as poorer quality of life, aligning with general long COVID epidemiology [5,7]. Freitas et al. also observed that poorer overall diet quality was associated with greater symptom burden, especially in women and in the domains of fatigue, memory, and mental health [12]. Furthermore, recent data have linked long COVID to heavier, prolonged, or intermenstrual bleeding and variations in symptomatic intensity throughout the cycle [49]. These results reinforce the need to consider menstrual losses and reproductive status when interpreting iron associations in women, though our interaction results should be interpreted cautiously due to reduced power in the male sample.

Crucially, dietary densities of iron and zinc were moderately correlated in women (Spearman’s ρ = 0.650). When both minerals were introduced simultaneously in the multivariable model, zinc maintained an inverse association with depressive symptoms after mutual adjustment, while the iron estimate was attenuated and lost statistical significance. Because collinearity was acceptable, this result mainly reflects the considerable variation shared between both exposures. This statistical dependence points toward common food sources or shared nutrient-dense dietary patterns. While zinc produced the most stable signal in the co-model, this alone does not identify it as the sole causative nutrient: correlation between exposures, dietary measurement error, and residual confounding can redistribute estimates when both minerals are evaluated simultaneously. A central finding of our exploratory analysis in women is that the inverse association between dietary iron and depressive symptoms was attenuated upon mutual adjustment with dietary zinc, whereas zinc density retained statistical significance. Rather than demonstrating an exclusive, independent biological effect of zinc over iron, this pattern most likely reflects the underlying dietary matrix. Iron and zinc share common dietary sources in high-quality diets; thus, the observed association may serve as a nutritional marker for broader dietary quality or a nutrient-dense dietary pattern rather than isolated single-mineral action.

### 4.3. Weak Nominal Signals—Iodine, Magnesium, and Sodium Associations

The remaining secondary associations observed in the unadjusted analyses represent weak, nominal signals that must be interpreted with high caution:

• Iodine and Mental Health-Related Quality of Life: Higher dietary iodine density was associated with a better score on the mental component of SF-36 in adjusted models and bootstrap analyses. This finding is of interest given the low proportion of participants meeting iodine recommendations [43]. However, the mental component of SF-36 is a broad indicator and does not reflect thyroid function. In a prospective follow-up of 250 survivors, most acute-phase thyroid abnormalities resolved, and neither thyroid function tests nor autoantibodies significantly predicted long COVID onset [50]. Therefore, this association should be considered strictly as a hypothesis-generating observation rather than evidence of a thyroid-mediated nutritional mechanism.
• Magnesium and Pain: The positive association found between dietary magnesium density and pain score was unexpected. Theoretically, magnesium modulates nociception via NMDA receptor regulation and neuromuscular excitability. While an inverse association between magnesium intake and chronic pain was observed in NHANES [51], a systematic review of randomized trials concluded that analgesic evidence was inconclusive [52]. The direction of our association is contrary to expected literature and should not be interpreted as causal. Magnesium density may function as a marker for specific dietary patterns; alternatively, reverse causality or chance in the context of multiple testing are plausible explanations. Furthermore, the absence of parallel associations with fatigue, sleep, or cognition—domains where magnesium has been previously suggested to play a role [13,14,15]—reinforces the need for cautious interpretation.
• Sodium Intake: Sodium adequacy represented adherence to the maximum intake limit (<2000 mg/day). Low adequacy reflected high prevalence of excess intake rather than insufficiency, showing no robust relationship with clinical outcomes after adjustment.

### 4.4. Clinical Implications

The results of the present study do not justify recommending iron, zinc, iodine, magnesium, or other mineral supplements as a treatment for long COVID. The observational and cross-sectional design prevents establishing causal relationships, and an intake lower than recommendations does not demonstrate a biochemical deficit. In addition, excessive administration of certain minerals can produce adverse effects. Although these findings do not demonstrate a direct therapeutic benefit of supplementation, they are compatible with the convenience of incorporating an individualized nutritional assessment into the multidimensional evaluation of patients with long COVID. This is particularly relevant when there are restrictive intakes, sustained alterations in taste or smell, weight loss, or other risk factors for malnutrition. Previous studies in post-COVID populations have shown a high frequency of nutritional risk and lower energy intake among patients with a higher symptom burden [9], and clinical reviews highlight the value of nutritional assessment while noting the absence of sufficient evidence to support specific mineral supplementation [8,11]. Identifying inappropriate dietary patterns and complementing the evaluation with objective biomarkers when indicated remains the most prudent clinical approach.

### 4.5. Strengths and Limitations

The present study has several strengths. First, dietary exposure was assessed using a detailed seven-day record and mineral intakes were expressed as density per 1000 kcal. This approach reduces the influence of total energy intake and characterizes the relative composition of the diet, although its coefficients should be interpreted as associations with a higher dietary concentration of the mineral and not with an isolated absolute increase in its intake. Second, nine minerals were simultaneously evaluated against an extensive clinical battery that included global burden of persistent symptoms, sleep, different dimensions of fatigue, subjective memory, objective cognitive function, frailty, physical and mental quality of life, anxiety, depression, pain, and dyspnea. This multidimensional characterization constitutes a relevant strength given the marked clinical heterogeneity of long COVID. Thirdly, the statistical analysis incorporated correction for multiple comparisons using FDR, multivariate models, sensitivity analysis using 5000 bootstrap samples, sex-stratified analyses and formal tests of mineral interaction × sex. In addition, for the most relevant associations, an additional model was carried out that simultaneously incorporated iron and zinc, which allowed assessing their statistical dependence within the same model, although not establishing causal independence.

However, several limitations should be recognized. First, the cross-sectional design precludes establishing temporality and causality. Persistent symptoms may modify food choices and dietary intake, so reverse causality is a possible explanation. Second, the large number of mineral–outcome comparisons increases the possibility of chance findings, particularly for analyses not corrected for multiplicity. Although False Discovery Rate (FDR) correction was applied to correlation screenings, no overall association remained significant after correction. Consequently, subsequent multivariate analyses should be considered exploratory and hypothesis-generating. Furthermore, because multivariate and bootstrap analyses were performed on mineral–outcome pairs preselected after nominal screening, post-selection inference limitations must be acknowledged. Standard inference and bootstrap resampling applied to preselected models do not fully account for the data-driven selection process itself; thus, bootstrap confidence intervals serve as metrics of internal stability rather than formal confirmatory inference. Similarly, formal sex-based interaction tests were not adjusted for multiplicity and the smaller male subsample reduced statistical power for sex-specific analyses, and absence of an association in men should not itself be interpreted as evidence of a female-specific effect. Third, dietary intake does not provide direct information on serum or tissue concentrations, bioavailability, intestinal absorption, or inflammation-associated mineral redistribution. Therefore, the classification of inadequate intake cannot be interpreted as equivalent to a mineral deficit. Fourth, although the seven-day dietary record provides a detailed characterization of usual intake, all dietary assessment methods are subject to recording errors and intra-individual variability. In addition, the density of a mineral may partially act as a marker of certain food sources or of an overall dietary pattern, especially when different micronutrients come from similar foods. This aspect is particularly relevant for interpreting the high correlation observed between iron and zinc densities. Fifth, residual and unmeasured confounding cannot be fully excluded, including socioeconomic characteristics, medication and supplement use, overall dietary patterns, and other behavioral or clinical factors potentially related to both mineral intake and psychological outcomes. Sixth, the smaller male sample size (*n* = 93 men vs. *n* = 197 women) reduced statistical power to detect subgroup-specific associations and formal interaction effects in men, adding uncertainty to male estimates. Additionally, interaction tests (k = 2) were unadjusted for multiple comparisons, which increases the possibility of chance findings and precludes definitive conclusions regarding sex dimorphism. This difference could have partially contributed to the observed pattern of modification by sex. In addition, variables such as menopausal status, menstrual losses or certain indicators of iron metabolism would have been especially relevant to interpret iron-related associations in women and should be incorporated into future research. Finally, the participants came from a single Spanish cohort of adults with established long COVID. Therefore, the generalization of the results to other populations, dietary contexts, evolutionary phases of the disease or individuals without long COVID should be carried out with caution.

## 5. Conclusions

In adults with long COVID, usual dietary mineral density did not show a robust global association with multidimensional clinical burden after FDR correction. However, several exploratory associations were identified that merit further investigation. The inverse associations identified between iron and zinc densities and depressive symptoms in women, along with their nominal interaction tests (k = 2), are exploratory and tentative. Due to the considerable correlation between the two exposures, the mutually adjusted model showed that zinc maintained an inverse association, while iron estimation was attenuated. These findings do not establish causality or support mineral supplementation, but they do highlight potential nutritional and psychological links that warrant verification in prospective, sex-balanced studies. In addition, higher iodine density was associated with better mental health-related quality of life, while the positive association found between magnesium and pain was unexpected and requires a particularly cautious interpretation. Taken together, these findings suggest that the intake of certain minerals could be related to specific dimensions of the heterogeneous phenotype of long COVID, particularly to psychological outcomes. However, the results do not establish causality or support the use of mineral supplements as treatment.

## 6. Future Perspectives

Future research should prospectively evaluate the relationship between dietary mineral intake and Long COVID by combining repeated longitudinal dietary assessments with circulating or functional biomarkers of mineral status. Larger multicenter cohorts featuring a balanced representation of women and men will be crucial to determine whether the sex-stratified findings observed here are reproducible. Finally, future analytical models should integrate comprehensive evaluations of overall dietary patterns, supplement use, medication exposure, inflammatory and metabolic biomarkers, socioeconomic factors, and relevant hormonal or reproductive variables. Particular attention may be warranted regarding the relationships observed between dietary iron and zinc densities and depressive symptoms; however, future research should determine whether these associations reflect specific biological effects of individual minerals or broader dietary patterns and correlated nutrient exposures. Longitudinal studies are strictly required to establish temporal relationships, and properly designed intervention trials would ultimately be necessary before recommending any mineral supplementation or targeted dietary strategy for individuals with Long COVID.

## Figures and Tables

**Table 1:** Characteristics of the analytical sample and dietary mineral density in the sample as a whole and according to sex.

	Total (*n* = 290)	Men (*n* = 93)	Women (*n* = 197)	*p* Value
Sociodemographic and Long COVID Characteristics				
Age, years	53.21 ± 11.71	56.23 ± 12.23	51.79 ± 11.21	0.002
Time since SARS-CoV-2 infection, months	40.23 [34.58–46.52]	40.70 [33.68–46.82]	40.13 [35.20–45.80]	0.652
Hospitalization during the acute phase of COVID-19, *n* (%)	81 (27.9)	40 (43.0)	41 (20.8)	<0.001
Antidepressant medication use, *n* (%)	75 (25.8)	14 (13.6)	61 (31.4)	0.114
Cardiometabolic characteristics				
Body mass index, kg/m^2^	27.71 [23.69–31.24]	29.40 [25.92–32.45]	26.51 [22.79–30.86]	<0.001
Hypertension, *n* (%)	105 (36.2)	50 (53.8)	55 (27.9)	<0.001
Diabetes mellitus, *n* (%)	37 (12.8)	22 (23.7)	15 (7.6)	<0.001
Dyslipidemia, *n* (%)	194 (66.9)	70 (75.3)	124 (62.9)	0.037
Lifestyle and diet characteristics				
Current smoking, *n* (%)	14 (4.8)	6 (6.5)	8 (4.1)	0.388
Alcohol consumption, g/week	0.0 [0.0–36.3]	25.0 [0.0–97.5]	0.0 [0.0–20.0]	<0.001
Steps/day	6612 [4706–9252]	6037 [4707–8783] (*n* = 90)	7344 [4678–9442] (*n* = 192)	0.178
Physical activity, MET-min/week	3360 [1680–7320]	3360 [1740–6600]	2940 [1600–8160]	0.365
Sedentary time, min/week	2520 [1470–3360]	2520 [1680–3675]	2100 [1260–3360]	0.089
MEDAS Score	8 [7–9]	8 [6–9]	8 [7–9]	0.487
Energy intake, kcal/day	1730 [1390–2039]	1782 [1365–2050]	1693 [1397–2026]	0.760
Dietary Mineral Density				
Calcium, mg/1000 kcal	423.34 [357.82–515.21]	416.50 [370.23–479.71]	426.37 [353.07–519.55]	0.573
Iron, mg/1000 kcal	7.80 [7.04–8.54]	7.70 [7.02–8.64]	7.82 [7.04–8.53]	0.598
Iodine, µg/1000 kcal	53.73 [43.90–64.11]	53.60 [42.50–65.87]	54.20 [44.12–63.95]	0.976
Magnesium, mg/1000 kcal	163.67 [144.80–180.79]	159.37 [143.74–181.19]	164.76 [146.11–180.80]	0.331
Zinc, mg/1000 kcal	5.39 [4.82–6.03]	5.32 [4.74–6.02]	5.43 [4.83–6.04]	0.640
Selenium, µg/1000 kcal	55.17 [46.67–66.20]	59.16 [46.72–71.71]	53.58 [46.61–64.57]	0.062
Sodium, mg/1000 kcal	1640 [1420–1884]	1675 [1439–1927]	1612 [1409–1871]	0.171
Potassium, mg/1000 kcal	1804.99 ± 315.67	1804.85 ± 323.89	1805.06 ± 312.56	0.996
Phosphorus, mg/1000 kcal	234.48 [183.38–294.56]	244.12 [197.20–298.58]	231.31 [177.96–289.75]	0.155

Data are presented as mean ± standard deviation, median [interquartile range], or *n* (%) as appropriate. Comparisons between sexes were performed using Student’s *t*-test for approximately normal variables, the Mann–Whitney U test for continuous non-normal variables, and Pearson’s chi-square test for categorical variables. Fisher’s exact test was used for current smoking because an expected frequency was <5. *p* values correspond to comparisons between men and women. Abbreviations: MEDAS, Mediterranean Diet Adherence Screener; MET, metabolic equivalent of the task.

**Table 2:** Clinical characteristics of the analytical sample as a whole and by sex.

	Total (*n* = 290)	Men (*n* = 93)	Women (*n* = 197)	*p* Value
Global Symptom Burden				
Number of persistent symptom domains	4 [3–5]	3 [2–5]	4 [3–6]	<0.001
Sleep and fatigue				
PSQI Total Score	10 [7–13]	8 [5.25–12]	10 [8–14]	<0.001
MFIS Physical Subscale	21 [11–28]	16 [7–24]	22 [13–29]	<0.001
MFIS Cognitive Subscale	19 [7–29]	14 [4–26]	23 [11.25–30.75]	0.001
MFIS psychosocial subscale	4 [1–6]	2 [0–5]	4 [2–6]	0.001
MFIS Total Score	45 [20–62]	32 [14–53]	48 [27–66]	<0.001
Cognition and memory				
Total score MFE-30	31 [16–53]	24.5 [13.25–42]	36 [18–55.5]	0.002
MoCA Total Score	24 [21–26]	23 [21–25]	24 [22–26]	0.116
Performance status and health-related quality of life				
FRAIL Score	1 [0–2]	0 [0–1]	1 [0–2]	<0.001
Physical summary component of the SF-36 (PCS)	40.53 [31.91–48.79]	45.55 [35.48–50.67]	38.45 [31.44–46.59]	0.001
SF-36 Mental Summary Component (MCS)	44.54 [30.62–53.44]	46.76 [34.26–54.06]	43.36 [29.07–52.41]	0.022
SF-36 Mental Health domain	62 [48–80]	64 [52–80]	60 [44–76]	0.010
Psychological symptoms				
GADS Anxiety Score	4 [1–7]	1 [0–6]	5 [1–7]	<0.001
GADS Depression Score	4 [1–6]	2 [0–5]	4 [2–6]	<0.001
Pain and shortness of breath				
Pain Score	27.5 [16–45.25]	19 [9–36]	32 [20–48]	<0.001
mMRC Dyspnea Score	1 [1–1]	1 [0–1]	1 [1–1]	0.026

Data are presented as median [interquartile range]. Sex-to-sex comparisons were performed using the Mann–Whitney U test. The number of available observations varied slightly between variables due to data absent on some clinical scales (total *n* = 281–290; men *n* = 90–93; women *n* = 190–197). Abbreviations: GADS, Goldberg Anxiety and Depression Scale; MCS, Mental Component Summary; MFE-30, Memory Failures of Everyday Questionnaire; MFIS, Modified Fatigue Impact Scale; mMRC, modified Medical Research Council; MoCA, Montreal Cognitive Assessment; PCS, Physical Component Summary; PSQI, Pittsburgh Sleep Quality Index; SF-36, 36-Item Short Form Health Survey.

**Table 3:** Sex-specific associations of dietary iron and zinc densities with depression score and formal interaction with sex.

Mineral	Men: B (95%CI)	*p*	Women: B (95%CI)	*p*	B Interaction	95% CI BCa	*p* Bootstrap	ΔR^2^	*p* ΔR^2^
Iron	0.044 (−0.467 to 0.554)	0.867	−0.591 (−0.945 to −0.236)	0.001	−0.634	−1.235 to −0.057	0.041	0.013	0.046
Zinc	0.183 (−0.373 to 0.739)	0.517	−0.653 (−0.993 to −0.312)	<0.001	−0.836	−1.488 to −0.222	0.015	0.019	0.013

The values are non-standardized regression coefficients (B) for a 1 standard deviation increase in dietary mineral density. The models were adjusted for age, time since SARS-CoV-2 infection, body mass index, and hospitalization during the acute phase. Men were the reference category; therefore, the interaction coefficient represents the difference in slope of women relative to men. Bootstrap estimates were based on 5000 resamplings and used 95% BCa confidence intervals. ΔR^2^ represents the additional variance explained by adding the mineral term × sex. The VIFs of the interaction terms were 3.12 for iron and 3.75 for zinc. The interaction *p* values were not adjusted for multiplicity. Abbreviations: BCa, bias-corrected and accelerated; CI, confidence interval; VIF, variance inflation factor.

## Data Availability

Data supporting the findings of this study are available at ZENODO: https://doi.org/10.5281/zenodo.14282873.

## Supplementary Materials

The following supporting information can be downloaded at: https://www.mdpi.com/article/10.3390/nu18203383/s1, Table S1. STROBE checklist for cross-sectional studies; Table S2. Cut-off points used to classify participants according to whether they met the recommended daily mineral intake; Table S3. Distributional assessment of continuous variables included in the mineral–clinical analyses; Table S4. Comparison of baseline characteristics between included (*n* = 290) and excluded (*n *= 14) participants; Table S5. Spearman correlations between dietary mineral density and clinical outcomes in the overall analytical sample; Table S6. Bootstrap sensitivity analyses for the selected fully adjusted mineral–clinical associations; Table S7. Expanded multivariable sensitivity analysis using ordinal logistic regression (PLUM) evaluating the independence of associations between dietary mineral densities and depressive symptoms (GADS); Figure S1. Percentage of participants meeting mineral intake recommendations in the overall sample and by sex; Figure S2. Sex-stratified Spearman correlations between dietary mineral density and clinical characteristics; Figure S3. Associations between dietary mineral intake density and psychological and pain outcomes.

## Abbreviations

BCa, Bias-corrected and accelerated confidence interval; CPGQ, Chronic Pain Grade Questionnaire; EFSA, European Food Safety Authority; FDR, false discovery rate; GADS, Goldberg Anxiety and Depression Scale; BMI, Body Mass Index; MCS, Mental Component Summary; MEDAS, Mediterranean Diet Adherence Screener; MET, Metabolic Equivalent of the Task; MFE-30, Memory Failures of Everyday Questionnaire de 30 items; MFIS, Modified Fatigue Impact Scale; mMRC, modified Medical Research Council; MoCA, Montreal Cognitive Assessment; PCS, Physical Component Summary; PSQI, Pittsburgh Sleep Quality Index; REDCap, Research Electronic Data Capture; SENC, Spanish Society of Community Nutrition; SF-36, 36-Item Short Form Health Survey; STROBE, Strengthening the Reporting of Observational Studies in Epidemiology; VIF, variance inflation factor; Ca, Calcium; Fe, Iron; I, iodine; K, potassium; Mg, magnesium; Na, sodium; P, Phosphorus; Se, selenium; Zn, zinc.
