# Supplementary material for: DietaryMineral Density and Clinical Outcomes in Adults with Long COVID: A Cross-Sectional Analysis of Global and Sex-Specific Associations in the BioICOPER Study
Source: Nutrients. 2026 Oct 15;18(20):3383. doi: 10.3390/nu18203383 (PMC13708554; doi:10.3390/nu18203383)
Supplement: Supplementary file 1 [file nutrients-18-03383-s001.zip › nutrients-4608111-supplementary.pdf]

Supplementary Table S1. STROBE checklist for cross-sectional studies

Section/topic	Item	STROBE recommendation (adapted)	Location in manuscript
Title and abstract	1a	Identify the cross-sectional design in the title or abstract.	Abstract; Section 2.1
Title and abstract	1b	Provide a balanced summary of methods and main findings.	Abstract
Background/rationale	2	Explain the scientific background and rationale.	Introduction, paragraphs 1–5
Objectives	3	State specific objectives and any prespecified hypotheses.	End of Introduction
Study design	4	Present the main design features early in the Methods.	Section 2.1
Setting	5	Describe setting, locations and relevant study dates.	Section 2.1 (recruitment during 2023)
Participants	6a	Report eligibility criteria and sources/methods of participant selection.	Section 2.1; Figure 1
Participants	6b	Matching criteria, when applicable.	Not applicable
Variables	7	Define outcomes, exposures, confounders and effect modifiers.	Sections 2.3–2.6
Data sources/measurement	8	Describe data sources and measurement methods for variables of interest.	Sections 2.2–2.5
Bias	9	Describe measures used to limit potential sources of bias.	Sections 2.2–2.4; Section 4.7
Study size	10	Explain how the analytical sample size was obtained.	Section 2.1 original sample-size plan in protocol [22]
Quantitative variables	11	Explain how quantitative variables were handled or categorized.	Sections 2.3 and 2.6; Table S2
Statistical methods	12a	Describe statistical methods, including confounding control.	Section 2.6
Statistical methods	12b	Describe subgroup and interaction analyses.	Section 2.6
Statistical methods	12c	Explain the handling of missing data.	Section 2.6
Statistical methods	12d	Describe methods accounting for the sampling strategy, if applicable.	Not applicable; consecutive sampling
Statistical methods	12e	Describe sensitivity analyses.	Section 2.6; bootstrap analyses
Participants	13a	Report numbers at each stage and the final number analysed.	Figure 1; Section 3.1
Participants	13b	Give reasons for non-participation or exclusion.	Figure 1
Participants	13c	Use a flow diagram when useful.	Figure 1
Descriptive data	14a	Report participant characteristics, exposures and relevant covariates.	Tables 1–2; Sections 3.1–3.2
Descriptive data	14b	Indicate missing observations for variables of interest.	Table 1; Table 2 note; Table S3
Descriptive data	14c	Summarize follow-up time for cohort studies, if relevant.	Not applicable to cross-sectional design
Outcome data	15	Report outcome summary measures.	Table 2
Main results	16a	Report unadjusted and adjusted estimates with precision and specify covariates.	Sections 3.3–3.5; Figures 2–4; Table 3
Main results	16b	Report cut-points when continuous variables are categorized.	Section 2.3; Table S2
Main results	16c	Translate relative risks to absolute risks when relevant.	Not applicable
Other analyses	17	Report subgroup, interaction and sensitivity analyses.	Sections 3.4–3.5; Figure S2; Table S5
Key results	18	Summarize key results in relation to the study objectives.	Opening of Discussion
Limitations	19	Discuss limitations, bias and imprecision.	Section 4.7

Section/topic	Item	STROBE recommendation (adapted)	Location in manuscript
Interpretation	20	Give a cautious interpretation considering multiplicity and other evidence.	Sections 4.1–4.7
Generalisability	21	Discuss external validity/generalizability.	Section 4.7
Funding	22	Report funding sources and the role of funders.	Funding statement

Table S2. Cut-off points used to classify participants according to whether they met the recommended daily mineral intake

**Mineral**	**Unit**	**Men Meeting**	**Men Not meeting**	**Women Meeting**	**Women Not meeting**
Calcium (Ca)	mg/day	≥1,000	<1,000	≥1,000	<1,000
Iron (Fe)	mg/day	≥10	<10	≤50 years ≥18<br>>50 years ≥10	≤50 years <18<br>>50 years <10
Iodine (I)	µg/day	≥150	<150	≥150	<150
Magnesium (Mg)	mg/day	≥350	<350	≥300	<300
Zinc (Zn)	mg/day	≥11	<11	≥8	<8
Selenium (Se)	µg/day	≥70	<70	≥70	<70
Sodium (Na)	mg/day	<2,000	≥2,000	<2,000	≥2,000
Potassium (K)	mg/day	≥3,500	<3,500	≥3,500	<3,500
Phosphorus (P)	mg/day	≥550	<550	≥550	<550

Cut-off points were established according to the recommendations of the Federación Española de Sociedades de Nutrición, Alimentación y Dietética (FESNAD). For all minerals except sodium, participants with intakes equal to or above the specified cut-off were classified as meeting the recommendation. For sodium, an intake below 2,000 mg/day was considered to meet the recommendation.

Reference. Federación Española de Sociedades de Nutrición, Alimentación y Dietética (FESNAD). Ingestas Dietéticas de Referencia (IDR) para la población española. Act Diet. 2010; 14(4): 196–197.

Supplementary Table S3. Distributional assessment of continuous variables included in the mineral–clinical analyses.

Variable	Skewness	Kurtosis	K–S D (p)	S–W W (p)	Presentation
Time since COVID-19, months	-1.060	0.889	0.135 (<0.001)	0.919 (<0.001)	Median [IQR]
Age, years	-0.246	-0.126	0.052 (0.057)	0.993 (0.194)	Mean ± SD
Body mass index, kg/m²	0.635	0.233	0.053 (0.047)	0.972 (<0.001)	Median [IQR]
MEDAS score	-0.765	1.165	0.148 (<0.001)	0.950 (<0.001)	Median [IQR]
Alcohol intake, g/week	2.719	8.964	0.291 (<0.001)	0.619 (<0.001)	Median [IQR]
Steps/day	0.847	0.437	0.088 (<0.001)	0.949 (<0.001)	Median [IQR]
Physical activity, MET-min/week	1.572	2.417	0.186 (<0.001)	0.827 (<0.001)	Median [IQR]
Sedentary time, min/week	0.876	1.131	0.117 (<0.001)	0.949 (<0.001)	Median [IQR]
Energy intake, kcal/day	0.808	1.362	0.053 (0.048)	0.966 (<0.001)	Median [IQR]
Calcium, mg/1000 kcal	0.965	3.451	0.056 (0.030)	0.956 (<0.001)	Median [IQR]
Iron, mg/1000 kcal	0.843	2.421	0.068 (0.003)	0.963 (<0.001)	Median [IQR]
Iodine, µg/1000 kcal	1.068	1.478	0.082 (<0.001)	0.934 (<0.001)	Median [IQR]
Magnesium, mg/1000 kcal	0.589	0.974	0.057 (0.026)	0.979 (<0.001)	Median [IQR]
Zinc, mg/1000 kcal	0.748	1.735	0.052 (0.053)	0.971 (<0.001)	Median [IQR]
Selenium, µg/1000 kcal	0.533	0.677	0.060 (0.013)	0.982 (0.001)	Median [IQR]
Sodium, mg/1000 kcal	0.854	0.962	0.098 (<0.001)	0.954 (<0.001)	Median [IQR]
Potassium, mg/1000 kcal	0.188	-0.238	0.029 (≥0.200)	0.994 (0.353)	Mean ± SD
Phosphorus, mg/1000 kcal	0.730	0.758	0.068 (0.002)	0.969 (<0.001)	Median [IQR]
Number of persistent symptom domains	-0.488	-0.806	0.172 (<0.001)	0.908 (<0.001)	Median [IQR]
PSQI total score	0.280	-0.552	0.088 (<0.001)	0.979 (<0.001)	Median [IQR]
MFIS physical subscale	-0.192	-1.096	0.078 (<0.001)	0.953 (<0.001)	Median [IQR]
MFIS cognitive subscale	-0.052	-1.286	0.105 (<0.001)	0.939 (<0.001)	Median [IQR]
MFIS psychosocial subscale	0.084	-1.207	0.111 (<0.001)	0.919 (<0.001)	Median [IQR]
MFIS total score	-0.112	-1.171	0.077 (<0.001)	0.954 (<0.001)	Median [IQR]
FRAIL total score	0.778	0.121	0.233 (<0.001)	0.840 (<0.001)	Median [IQR]
MFE-30 total score	0.606	-0.216	0.094 (<0.001)	0.956 (<0.001)	Median [IQR]
SF-36 PCS	-0.285	-0.622	0.056 (0.035)	0.981 (<0.001)	Median [IQR]
SF-36 MCS	-0.376	-1.008	0.107 (<0.001)	0.946 (<0.001)	Median [IQR]

Variable	Skewness	Kurtosis	K–S D (p)	S–W W (p)	Presentation
SF-36 mental health domain	-0.328	-0.264	0.087 (<0.001)	0.978 (<0.001)	Median [IQR]
GADS anxiety score	0.073	-1.581	0.231 (<0.001)	0.860 (<0.001)	Median [IQR]
GADS depression score	−0.076	−1.224	0.152 (<0.001)	0.914 (<0.001)	Median [IQR]
CPGQ pain score	0.179	-1.025	0.089 (<0.001)	0.965 (<0.001)	Median [IQR]
MoCA total score	-0.710	0.936	0.109 (<0.001)	0.963 (<0.001)	Median [IQR]

K–S, Kolmogorov–Smirnov test with Lilliefors correction; S–W, Shapiro–Wilk test. Age and potassium density were approximately normally distributed and are presented as mean ± standard deviation; the remaining continuous or bounded variables are presented as median [interquartile range]. The ordinal mMRC dyspnea score was not included in the distributional assessment. Abbreviations: CPGQ, Chronic Pain Grade Questionnaire; GADS, Goldberg Anxiety and Depression Scale; IQR, interquartile range; MCS, Mental Component Summary; MEDAS, Mediterranean Diet Adherence Screener; MET, metabolic equivalent of task; MFIS, Modified Fatigue Impact Scale; MoCA, Montreal Cognitive Assessment; PCS, Physical Component Summary; PSQI, Pittsburgh Sleep Quality Index; SD, standard deviation; SF-36, 36-Item Short Form Health Survey.

Supplementary Table S4. Comparison of baseline characteristics between included ($n = 290$) and excluded ($n = 14$) participants.

Characteristic	Included Participants (n=290)	Excluded Participants (n=14)	p-value
**Sociodemographic and Clinical Characteristics**			
Age, years (mean ± SD)	53.21 ± 11.71	43.79 ± 12.91	0.004
Time since SARS-CoV-2 infection, months (median [IQR])	40.23 [34.58–46.52]	40.00 [35.00–47.00]	0.962
Hospitalization during acute COVID-19, n (%)	81 (27.9)	1 (7.1)	0.277
Antidepressant medication use, n (%)	75 (25.8)	1 (7.1)	0.077
**Cardiometabolic Characteristics**			
Body mass index, kg/m² (mean ± SD)	28.06 ± 5.52	26.52 ± 6.17	0.310
Hypertension, n (%)	105 (36.2)	4 (28.6)	0.561
Diabetes mellitus, n (%)	37 (12.8)	0 (0.0)	0.232
Dyslipidemia, n (%)	194 (66.9)	7 (50.0)	0.192
**Lifestyle Characteristics**			
Mediterranean Diet Score (0–14 points) (mean ± SD)	7.80 ± 2.31	7.79 ± 3.02	0.982
Alcohol consumption, g/week (mean ± SD)	29.54 ± 53.66	24.64 ± 35.76	0.447
Current smoking, n (%)	14 (4.8)	2 (14.3)	0.163
Physical activity, steps/day (mean ± SD)*	7409.24 ± 3730.99	8369.96 ± 2639.89	0.379
Physical activity, METs-min/week (mean ± SD)	5094.00 ± 4961.26	5875.71 ± 5961.89	0.568

SD: Standard Deviation; IQR: Interquartile Range. Continuous variables were compared using Student's t-test or Mann-Whitney U test as appropriate. Categorical variables were compared using the Chi-square test.

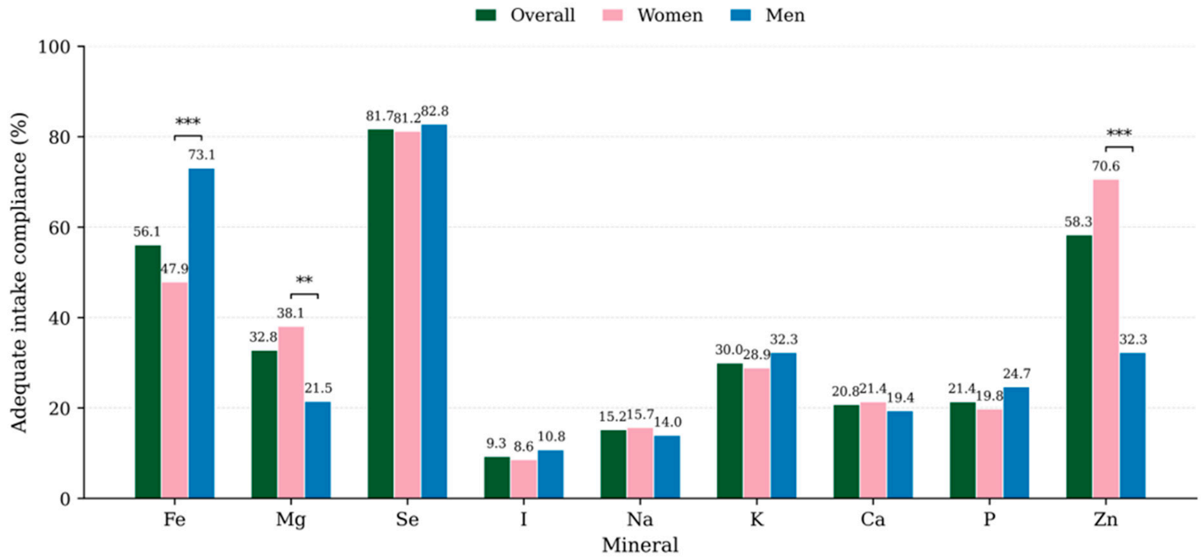

Supplementary Figure S1. Percentage of participants meeting mineral intake recommendations in the overall sample and by sex. Bars represent the proportion of participants classified as having adequate intake for each mineral according to the study thresholds, using sex-specific cut-offs when applicable. Asterisks indicate differences between women and men (Pearson's chi-square test): ** $p < 0.01$; *** $p < 0.001$. Abbreviations: Ca, calcium; Fe, iron; I, iodine; K, potassium; Mg, magnesium; Na, sodium; P, phosphorus; Se, selenium; Zn, zinc.

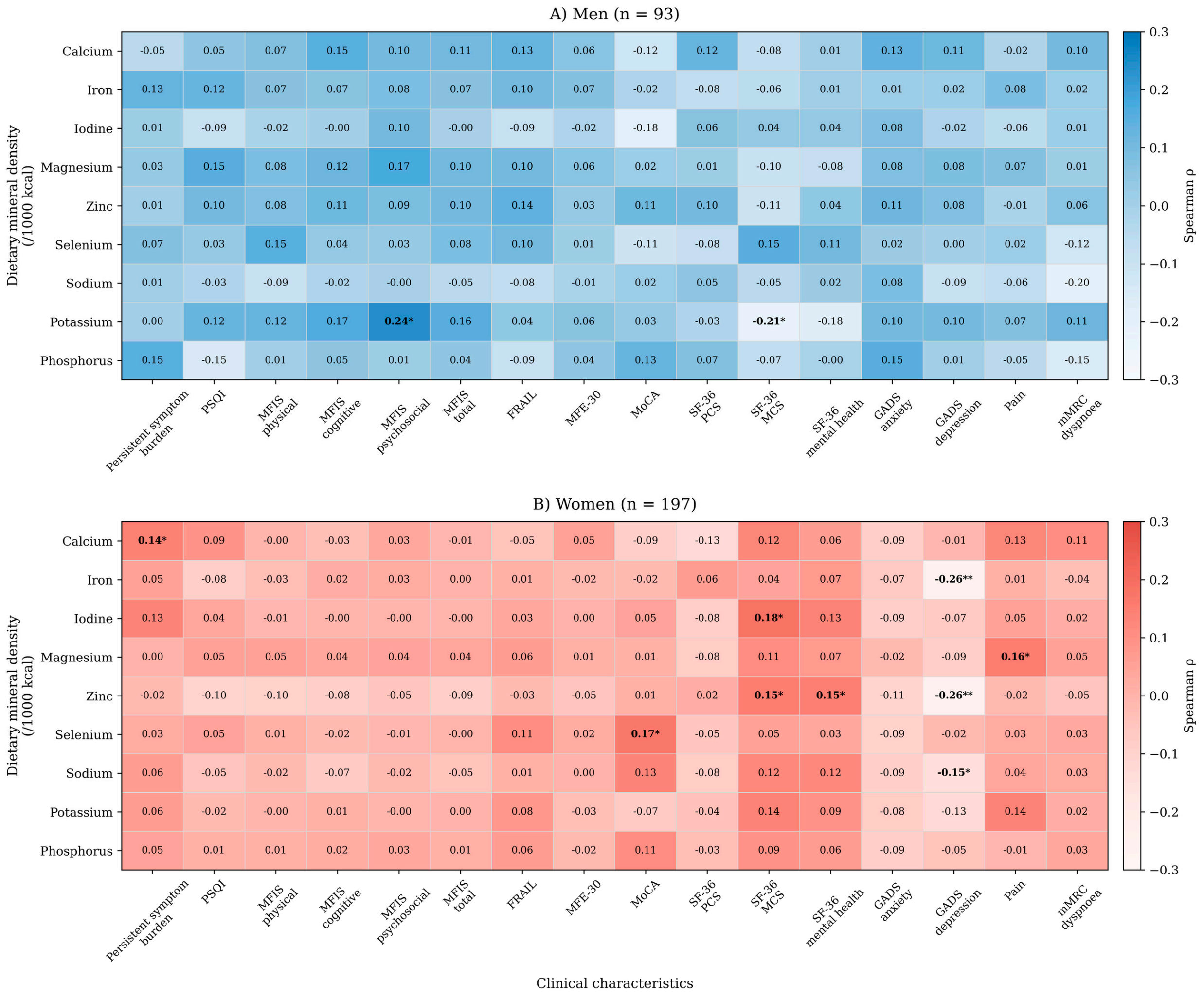

* p < 0.05 (nominal); ** q < 0.05 after Benjamini–Hochberg FDR correction across 288 sex-stratified tests.
Only iron–depression and zinc–depression in women remained significant after FDR correction.

Supplementary Figure S2. Sex-stratified Spearman correlations between dietary mineral density and clinical characteristics. Heatmaps display Spearman's rank correlation coefficients (ϱ) in men (n = 93) and women (n = 197). Mineral density was expressed per 1000 kcal. A single asterisk (*) indicates nominal p < 0.05, and a double asterisk (**) indicates q < 0.05 after Benjamini–Hochberg false discovery rate correction across the 288 sex-stratified correlations. Sample size varied slightly across clinical outcomes because of missing data.

Supplementary Table S5. Spearman correlations between dietary mineral density and clinical outcomes in the overall analytical sample.

Mineral	Clinical outcome	Spearman ρ	p-value	q-value (FDR)
**Calcium**	Number of persistent symptom domains	0.087	0.137	0.968
Calcium	PSQI total score	0.075	0.206	0.968
Calcium	MFIS physical	0.023	0.701	0.968
Calcium	MFIS cognitive	0.022	0.712	0.968
Calcium	MFIS psychosocial	0.060	0.313	0.968
Calcium	MFIS total score	0.030	0.615	0.968
Calcium	FRAIL total score	0.002	0.975	0.995
Calcium	MFE-30 total score	0.050	0.397	0.968
Calcium	MoCA total score	-0.091	0.125	0.968
Calcium	SF-36 PCS	-0.064	0.280	0.968
Calcium	SF-36 MCS	0.066	0.270	0.968
Calcium	SF-36 Mental Health	0.037	0.535	0.968
Calcium	GADS anxiety	-0.033	0.575	0.968
Calcium	GADS depression	0.039	0.505	0.968
Calcium	CPGQ pain score	0.078	0.194	0.968
Calcium	mMRC dyspnea	0.099	0.098	0.968
**Iron**	Number of persistent symptom domains	0.092	0.116	0.968
Iron	PSQI total score	-0.001	0.986	0.995
Iron	MFIS physical	0.021	0.728	0.968
Iron	MFIS cognitive	0.046	0.443	0.968
Iron	MFIS psychosocial	0.057	0.340	0.968
Iron	MFIS total score	0.041	0.496	0.968
Iron	FRAIL total score	0.045	0.445	0.968
Iron	MFE-30 total score	0.028	0.642	0.968
Iron	MoCA total score	-0.017	0.768	0.968
Iron	SF-36 PCS	-0.001	0.981	0.995
Iron	SF-36 MCS	0.006	0.917	0.995
Iron	SF-36 Mental Health	0.047	0.431	0.968

Mineral	Clinical outcome	Spearman ρ	p-value	q-value (FDR)
Iron	GADS anxiety	-0.024	0.680	0.968
Iron	GADS depression	**-0.140**	**0.017**	0.752
Iron	CPGQ pain score	0.067	0.265	0.968
Iron	mMRC dyspnea	-0.016	0.793	0.977
**Iodine**	Number of persistent symptom domains	0.094	0.112	0.968
Iodine	PSQI total score	0.001	0.984	0.995
Iodine	MFIS physical	-0.005	0.935	0.995
Iodine	MFIS cognitive	0.006	0.925	0.995
Iodine	MFIS psychosocial	0.029	0.632	0.968
Iodine	MFIS total score	0.007	0.908	0.995
Iodine	FRAIL total score	-0.007	0.904	0.995
Iodine	MFE-30 total score	0.002	0.979	0.995
Iodine	MoCA total score	-0.022	0.711	0.968
Iodine	SF-36 PCS	-0.034	0.564	0.968
Iodine	SF-36 MCS	**0.137**	**0.021**	0.752
Iodine	SF-36 Mental Health	0.105	0.078	0.968
Iodine	GADS anxiety	-0.040	0.498	0.968
Iodine	GADS depression	-0.045	0.449	0.968
Iodine	CPGQ pain score	0.018	0.758	0.968
Iodine	mMRC dyspnea	0.017	0.773	0.968
**Magnesium**	Number of persistent symptom domains	0.038	0.518	0.968
Magnesium	PSQI total score	0.086	0.148	0.968
Magnesium	MFIS physical	0.073	0.220	0.968
Magnesium	MFIS cognitive	0.071	0.234	0.968
Magnesium	MFIS psychosocial	0.090	0.132	0.968
Magnesium	MFIS total score	0.076	0.200	0.968
Magnesium	FRAIL total score	0.078	0.187	0.968
Magnesium	MFE-30 total score	0.040	0.502	0.968
Magnesium	MoCA total score	0.013	0.825	0.992
Magnesium	SF-36 PCS	-0.063	0.295	0.968

Mineral	Clinical outcome	Spearman ρ	p-value	q-value (FDR)
Magnesium	SF-36 MCS	0.042	0.480	0.968
Magnesium	SF-36 Mental Health	0.024	0.689	0.968
Magnesium	GADS anxiety	0.022	0.709	0.968
Magnesium	GADS depression	-0.019	0.747	0.968
Magnesium	CPGQ pain score	**0.141**	**0.017**	0.752
Magnesium	mMRC dyspnea	0.035	0.556	0.968
**Zinc**	Number of persistent symptom domains	-0.004	0.949	0.995
Zinc	PSQI total score	-0.031	0.608	0.968
Zinc	MFIS physical	-0.035	0.557	0.968
Zinc	MFIS cognitive	-0.021	0.731	0.968
Zinc	MFIS psychosocial	-0.011	0.848	0.992
Zinc	MFIS total score	-0.024	0.684	0.968
Zinc	FRAIL total score	0.030	0.614	0.968
Zinc	MFE-30 total score	-0.026	0.668	0.968
Zinc	MoCA total score	0.047	0.424	0.968
Zinc	SF-36 PCS	0.042	0.481	0.968
Zinc	SF-36 MCS	0.075	0.209	0.968
Zinc	SF-36 Mental Health	0.113	0.058	0.968
Zinc	GADS anxiety	-0.039	0.508	0.968
Zinc	GADS depression	**-0.129**	**0.028**	0.804
Zinc	CPGQ pain score	-0.013	0.831	0.992
Zinc	mMRC dyspnea	-0.011	0.854	0.992
**Selenium**	Number of persistent symptom domains	0.017	0.773	0.968
Selenium	PSQI total score	0.007	0.908	0.995
Selenium	MFIS physical	0.033	0.579	0.968
Selenium	MFIS cognitive	-0.029	0.630	0.968
Selenium	MFIS psychosocial	-0.025	0.676	0.968
Selenium	MFIS total score	0.000	0.997	0.997
Selenium	FRAIL total score	0.068	0.248	0.968
Selenium	MFE-30 total score	-0.001	0.981	0.995

Mineral	Clinical outcome	Spearman ρ	p-value	q-value (FDR)
Selenium	MoCA total score	0.065	0.271	0.968
Selenium	SF-36 PCS	-0.036	0.546	0.968
Selenium	SF-36 MCS	0.103	0.083	0.968
Selenium	SF-36 Mental Health	0.077	0.197	0.968
Selenium	GADS anxiety	-0.085	0.147	0.968
Selenium	GADS depression	-0.038	0.525	0.968
Selenium	CPGQ pain score	0.008	0.899	0.995
Selenium	mMRC dyspnea	-0.049	0.415	0.968
**Sodium**	Number of persistent symptom domains	0.013	0.824	0.992
Sodium	PSQI total score	-0.058	0.330	0.968
Sodium	MFIS physical	-0.056	0.349	0.968
Sodium	MFIS cognitive	-0.064	0.281	0.968
Sodium	MFIS psychosocial	-0.031	0.608	0.968
Sodium	MFIS total score	-0.057	0.336	0.968
Sodium	FRAIL total score	-0.043	0.467	0.968
Sodium	MFE-30 total score	-0.018	0.764	0.968
Sodium	MoCA total score	0.086	0.148	0.968
Sodium	SF-36 PCS	-0.022	0.707	0.968
Sodium	SF-36 MCS	0.085	0.155	0.968
Sodium	SF-36 Mental Health	0.100	0.094	0.968
Sodium	GADS anxiety	-0.057	0.336	0.968
Sodium	GADS depression	**-0.136**	**0.020**	0.752
Sodium	CPGQ pain score	0.001	0.988	0.995
Sodium	mMRC dyspnea	-0.068	0.259	0.968
**Potassium**	Number of persistent symptom domains	0.053	0.364	0.968
Potassium	PSQI total score	0.020	0.740	0.968
Potassium	MFIS physical	0.047	0.427	0.968
Potassium	MFIS cognitive	0.067	0.261	0.968
Potassium	MFIS psychosocial	0.074	0.212	0.968
Potassium	MFIS total score	0.062	0.299	0.968

Mineral	Clinical outcome	Spearman ρ	p-value	q-value (FDR)
Potassium	FRAIL total score	0.064	0.278	0.968
Potassium	MFE-30 total score	0.008	0.892	0.995
Potassium	MoCA total score	-0.040	0.504	0.968
Potassium	SF-36 PCS	-0.046	0.439	0.968
Potassium	SF-36 MCS	0.033	0.576	0.968
Potassium	SF-36 Mental Health	0.011	0.849	0.992
Potassium	GADS anxiety	-0.021	0.722	0.968
Potassium	GADS depression	-0.051	0.386	0.968
Potassium	CPGQ pain score	0.116	0.051	0.968
Potassium	mMRC dyspnea	0.054	0.371	0.968
**Phosphorus**	Number of persistent symptom domains	0.052	0.378	0.968
Phosphorus	PSQI total score	-0.066	0.266	0.968
Phosphorus	MFIS physical	-0.011	0.854	0.992
Phosphorus	MFIS cognitive	0.019	0.746	0.968
Phosphorus	MFIS psychosocial	0.003	0.957	0.995
Phosphorus	MFIS total score	0.005	0.930	0.995
Phosphorus	FRAIL total score	-0.010	0.866	0.995
Phosphorus	MFE-30 total score	-0.016	0.794	0.977
Phosphorus	MoCA total score	0.103	0.083	0.968
Phosphorus	SF-36 PCS	0.020	0.736	0.968
Phosphorus	SF-36 MCS	0.054	0.361	0.968
Phosphorus	SF-36 Mental Health	0.058	0.333	0.968
Phosphorus	GADS anxiety	-0.033	0.577	0.968
Phosphorus	GADS depression	-0.042	0.475	0.968
Phosphorus	CPGQ pain score	-0.038	0.527	0.968
Phosphorus	mMRC dyspnea	-0.045	0.450	0.968

Mineral intake was expressed as dietary density per 1000 kcal. q-values were calculated using the Benjamini–Hochberg false discovery rate procedure across all 144 mineral–outcome correlations. Nominal p-values <0.05 are highlighted; none of the correlations remained significant after false discovery rate correction. Abbreviations: CPGQ, Chronic Pain Grade Questionnaire; FDR, false discovery rate; GADS, Goldberg Anxiety and Depression Scale; MCS, Mental Component Summary; MFE-30, Memory Failures of Everyday questionnaire; MFIS, Modified Fatigue Impact Scale; mMRC, modified Medical Research Council; MoCA, Montreal Cognitive Assessment; PCS, Physical Component Summary; PSQI, Pittsburgh Sleep Quality Index; SF-36, 36-Item Short Form Health Survey.

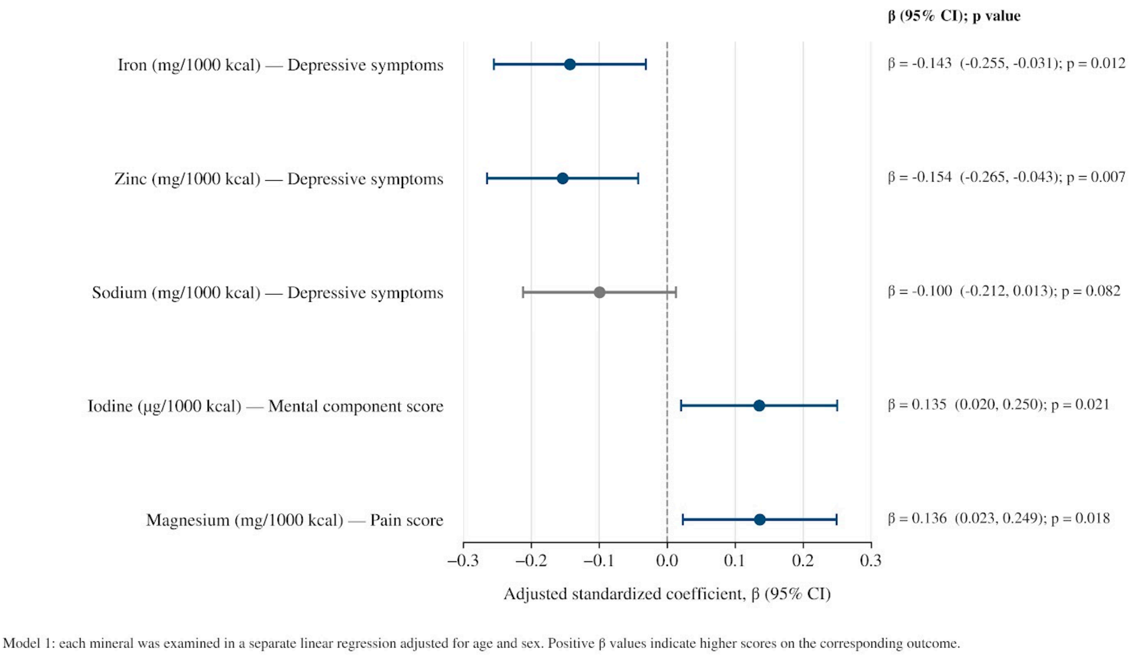

Figure S3. Associations between dietary mineral intake density and psychological and pain outcomes. Non-standardized regression coefficients (B) and their 95% confidence intervals are shown for the five mineral-clinical pairs selected after nominal screening. Model adjusted by time since SARS-CoV-2 infection, body mass index, hospitalization during the acute phase and total energy intake (kcal/day). Associations with iron, zinc, iodine, and magnesium maintained a similar direction and magnitude after additional adjustment, while the confidence interval of the sodium-depression association included the null value. These analyses are exploratory because no overall correlation exceeded FDR correction.

Supplementary Table S6. Bootstrap sensitivity analyses for the selected fully adjusted mineral–clinical associations.

Dietary mineral density	Clinical outcome	B	Bootstrap bias	Bootstrap SE	BCa 95% CI	p
Iron density, mg/1000 kcal	Depressive symptoms (GADS-Dep)	−0.296	−0.00009	0.107	**−0.506 to −0.078**	**0.005**
Zinc density, mg/1000 kcal	Depressive symptoms (GADS-Dep)	−0.450	0.00285	0.142	**−0.716 to −0.157**	**0.001**
Sodium density, mg/1000 kcal	Depressive symptoms (GADS-Dep)	−0.00060	−0.000013	0.00037	−0.00130 to 0.00008	0.103
Iodine density, μg/1000 kcal	SF-36 Mental Component Summary score	0.094	0.00028	0.042	**0.010 to 0.176**	**0.025**
Magnesium density, mg/1000 kcal	CPGQ pain score	0.086	0.00100	0.034	**0.020 to 0.155**	**0.010**

Models were adjusted for age, sex, time since SARS-CoV-2 infection, body mass index, and hospitalization during acute COVID-19. Bootstrap estimates were based on 5,000 resamples using bias-corrected and accelerated (BCa) 95% confidence intervals. Abbreviations: B, unstandardized regression coefficient; BCa, bias-corrected and accelerated; CI, confidence interval; CPGQ, Chronic Pain Grade Questionnaire; GADS-Dep, Goldberg Anxiety and Depression Scale depression score; SE, standard error; SF-36, 36-Item Short Form Health Survey.

Supplementary Table S7. Expanded multivariable sensitivity analysis using ordinal logistic regression (PLUM) evaluating the independence of associations between dietary mineral densities and depressive symptoms (GADS)

Mineral exposure	Sample	Model	B coefficient	95% CI	p-value
**Zinc density** *(mg/1000 kcal)*	**Total**	Model 2 (Previous adjustment) (n=290)	-0.344	-0.566 to -0.122	0.002
		**Model 3 (Expanded adjustment) (n=290)**	-0.442	-0.675 to -0.209	<0.001
	**Women**	Model 2 (Previous adjustment) **(n=197)**	-0.511	-0.780 to -0.243	<0.001
		**Model 3 (Expanded adjustment) (n=197)**	**-0.943**	**-1.493 to -0.392**	**0.001**
	**Men**	Model 2 (Previous adjustment) **(n=93)**	-0.592	-0.876 to -0.308	<0.001
		**Model 3 (Expanded adjustment) (n=93)**	0.116	-0.325 to 0.557	0.605
**Iron density** *(mg/1000 kcal)*	**Total**	Model 2 (Previous adjustment) (n=290)	0.061	-0.397 to 0.519	0.795
		**Model 3 (Expanded adjustment)** (n=290)	-0.235	-0.396 to -0.075	0.004
	**Women**	Model 2 (Previous adjustment) (n=197)	-0.293	-0.460 to -0.125	<0.001
		**Model 3 (Expanded adjustment)** (n=197)	-0.344	-0.544 to -0.145	<0.001
	**Men**	Model 2 (Previous adjustment) (n=93)	-0.438	-0.652 to -0.223	<0.001
		**Model 3 (Expanded adjustment)** (n=93)	-0.058	-0.352 to 0.236	0.700

Dependent variable: Depressive symptoms score from the Goldberg Anxiety and Depression Scale (GADS). All estimates are derived from **Ordinal Logistic Regression (PLUM - Polytomous Universal Model)** to account for the bounded and discrete distribution of the GADS score (0–9). Model 2 (Standard previous adjustment): Adjusted for age, sex (in total sample), time since SARS-CoV-2 infection (months), body mass index (BMI), and hospitalization during acute COVID-19. Model 3 (Expanded sensitivity adjustment): Adjusted for Model 2 covariates plus Mediterranean diet adherence (MEDAS score), total energy intake (kcal/day), alcohol consumption (g/week), and baseline antidepressant medication use (Yes/No). Abbreviations: B, unstandardized ordinal regression location coefficient; CI, confidence interval; GADS, Goldberg Anxiety and Depression Scale; MEDAS, Mediterranean Diet Adherence Screener; PLUM, Polytomous Universal Model (Ordinal Logistic Regression).
